# Xanthan Gum–Iron System: Natural, Mechanically Tunable, Bioactive, and Magnetic-Responsive Hydrogels for Biomedical Engineering Applications

**DOI:** 10.1021/acsami.5c08442

**Published:** 2025-09-03

**Authors:** Monize C. Decarli, Joanna Babilotte, Wen Chen, Julian Kappesz, Tim ten Brink, Lisanne Dechant, Maria Kalogeropoulou, Clarissa Tomasina, Catarina A. Custódio, João F. Mano, Lorenzo Moroni

**Affiliations:** † MERLN Institute for Technology-Inspired Regenerative Medicine, Department of Complex Tissue Regeneration, 5211 Maastricht University, Universiteitssingel 40, 6229 ER Maastricht, The Netherlands; ‡ Department of Biomaterials & Biomedical Technology, University Medical Center Groningen/University of Groningen, A. Deusinglaan 1, AV 9713 Groningen, The Netherlands; § CICECO—Department of Chemistry, Aveiro Institute of Materials, University of Aveiro, Campus Universitário de Santiago, 3810-193 Aveiro, Portugal; ∥ Metatissue, PCI, Creative Science Park Aveiro Region, Via do Conhecimento, 3830-352 Ílhavo, Portugal

**Keywords:** xanthan gum, 3D printing, hydrogel, ionic crosslinking, tissue engineering, biomedical engineering

## Abstract

Xanthan gum (XG) has performed far better than other polysaccharides for industrial purposes, e.g., food, pharmaceutical, and cosmetic applications, due to its outstanding thickening effect, pseudoplastic rheological properties, and non-toxicity. However, there is no crosslinking strategy available for non-modified XG that allows its sole use within cells for biomedical engineering applications. Here, we established this crosslinking strategy while processing it via additive manufacturing techniques. The suitability of divalent (Ca^2+^, Mg^2+^, and Fe^2+^) and trivalent (Al^3+^ and Fe^3+^) ions was evaluated by an in situ rheological assessment. Fe^3+^ demonstrated a high affinity to XG by forming a stable crosslinking effect, and the baseline XG–Fe^3+^ hydrogel exhibited outstanding printability and high culture stability (60 days). Although XG–Fe^3+^ demonstrated high biocompatibility for hMSCs with sustained cytocompatible iron release, these cell-laden constructs are inert. Envisioning biological functionality, we blended human methacryloyl platelet lysates (hPLMA) with XG–Fe^3+^ and either used inert XG–Fe^3+^ or bioactive cell-adhesive XG–Fe^3+^–PLMA, resulting in a 10-fold increase in strength compared to non-crosslinked XG. Remarkably, whether inert or bioactive, hydrogels proved to be mechanically tunable (from ∼3 to 203 kPa), ideal for tissue engineering applications. Later, we expanded the XG–Fe^3+^ role to a delivery system using magnetic nanoparticles (MNPs), and magnetically responsive scaffolds were obtained (XG–Fe^3+^–MNP). Finally, to explore the convergence of 3D printing and melt electrowriting (MEW), polycaprolactone (PCL) was included to obtain hybrid scaffolds (XG–PLMA–PCL). Our findings present a novel XG–Fe^3+^ hydrogel with remarkable versatility as a natural, mechanically tunable, bioactive, and magnetic- responsive system for sole or hybrid use. This unusual set of capabilities meets the current demand for developing tailored hydrogels for complex biomedical engineering applications.

## Graphical abstract

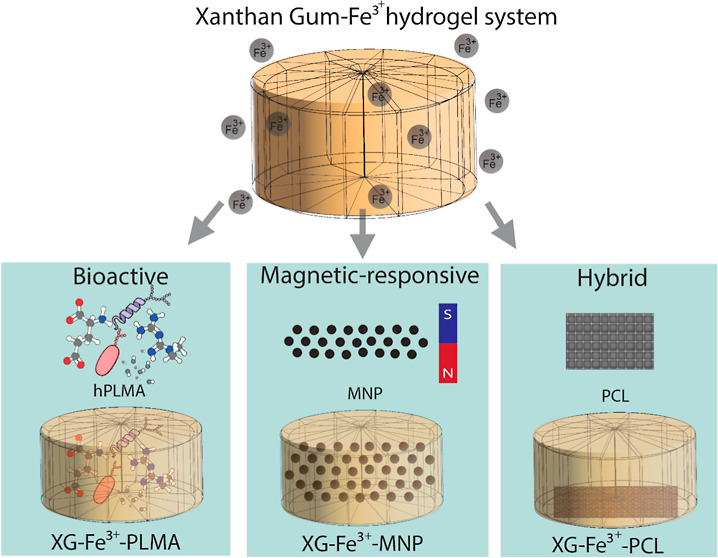

## Introduction

Hydrogels are crosslinked polymer chains that can form 3D networks with a high water content, tunable stiffness, and desired porosity that closely resemble extracellular matrices and fluid phases of our tissues and organs. Among hydrogels composed of natural polysaccharides, alginate is the most widely used material due to its biocompatibility, either as a scaffold and for cell encapsulation, while demonstrating an efficient ionic crosslinking by divalent ions.^1^ However, alginate has limitations, e.g., poor stability for long culture periods, low-medium viscosity that impacts its printability, and low mechanical properties.^1–3^ Overcoming these limitations, xanthan gum (XG) has been currently investigated as an alternative natural polysaccharide due to its high emulsion-stabilizing activity, pseudoplastic rheological properties, and high viscosities even at low concentrations.^3–5^ Due to its outstanding thickening effect, nontoxicity, and stability under different conditions of pH, temperature, and ionic concentrations,^6^ XG has been widely applied for industrial purposes (Figure 1A), e.g., food, pharmaceutical, cosmetics, oil-recovery, and petroleum industries.^7^ Although the advantages of XG are attractive, no crosslinking strategy is available for non-modified XG that supports its sole application within cells, enabling biomedical engineering applications.

**Figure 1:**
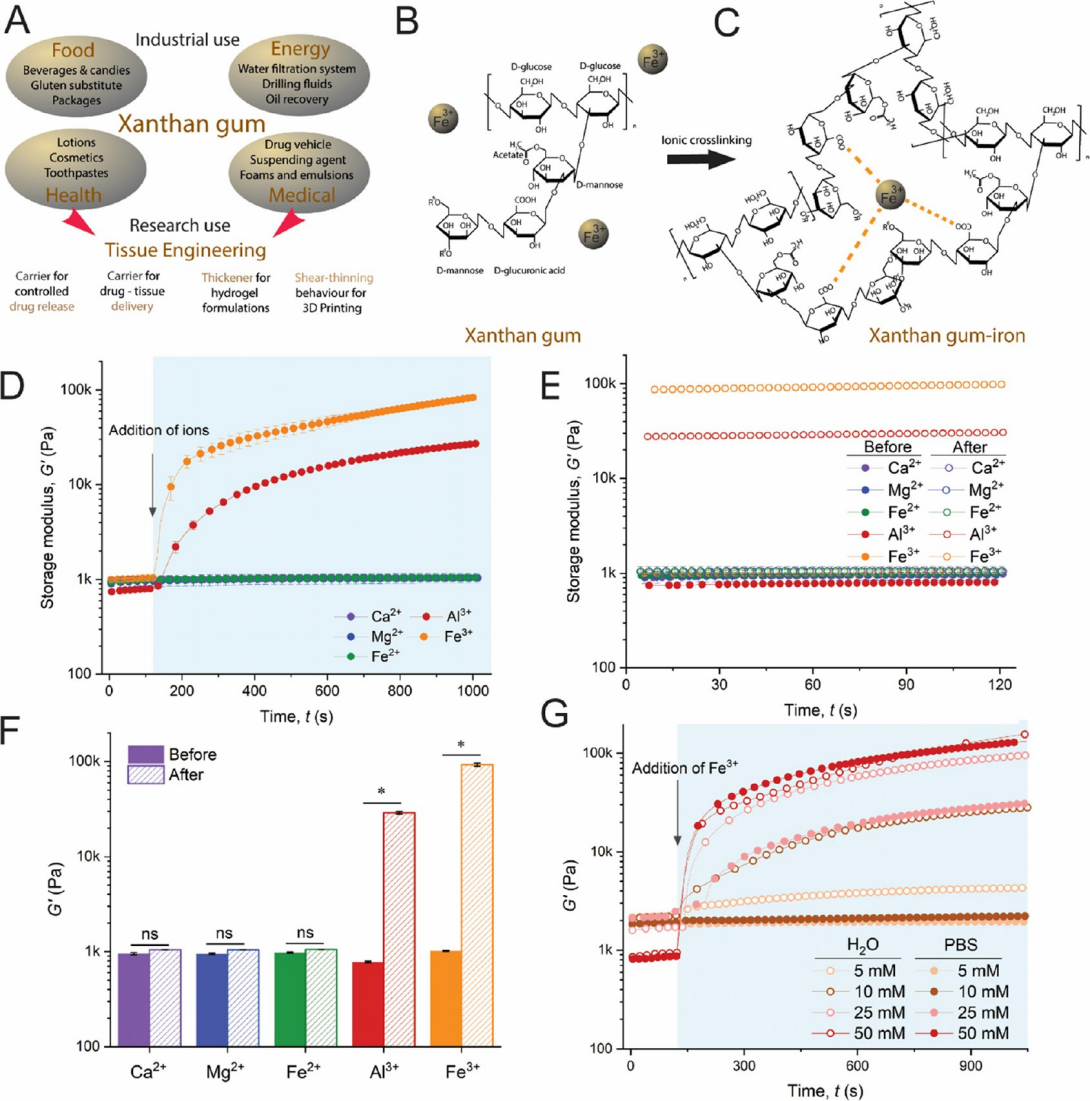
(A) The high industrial applicability of xanthan gum (XG) and the potential areas for translating the research studies of XG to tissue engineering applications in the clinics. (B) The structural form of XG and (C) a schematic illustration for crosslinking XG using Fe^3+^. Mechanical properties of XG in the presence of divalent and trivalent cations: (D) in situ ionic crosslinking effects of aqueous 50 mM Ca^2+^, Mg^2+^, Fe^2+^, Al^3+^, and Fe^3+^ on the storage modulus (*G*′) of 7 wt % XG hydrogel and (E) time sweeps of the storage modulus *G*′ of the XG hydrogel before and after aqueous 50 mM Ca^2+^, Mg^2+^, Fe^2+^, Al^3+^, and Fe^3+^ addition using a rheometer equipped with a homemade cup-shaped accessory. (F) Storage modulus of the XG hydrogel before and after aqueous Ca^2+^, Mg^2+^, Fe^2+^, Al^3+^, and Fe^3+^ addition, using a rheometer equipped with a 1000 μm geometry gap accessory (significant differences *, *p* ≤ 0.05; ns, not significant). (G) Mechanical properties of XG ranging from low to high Fe^3+^ concentrations dissolved in water and in PBS to mimic the conditions when doing cellular culture experiments, using a rheometer equipped with a 500 μm geometry gap accessory.

To date, several crosslinking strategies have been developed for XG. First, due to high XG functionality, particularly in challenging industrial environments, crosslinking strategies based on chromium chloride,^8^ epichlorohydrin,^9^ sodium trimetaphosphate,^10^ glutaraldehyde,^10^ and citric acid via β-cyclodextrin^11^ were developed. However, these agents are mostly toxic to cells or release harmful residues even at low concentrations and thus cannot be used for biomedical engineering purposes. An exception is presented by crosslinking via citric acid, which could be performed in non-toxic conditions. However, the process requires a high temperature (165 °C),^11^ which is not tolerated by cells. Interestingly, when using sodium trimetaphosphate and glutaraldehyde, biocompatibility tests demonstrated the high viability of cancer cells. Yet, XG was not solely used in the study; a mixture of gellan gum, XG, poly­(vinyl alcohol), and hydroxyapatite was used, which hindered the solely XG crosslinking effect. Lastly, for food and pharmaceutical industries, XG crosslinking was also pursued by employing natural deep eutectic solvents (NADES), as a green alternative, but the obtained hydrogels were weak.^12^

Another commonly explored route to crosslink XG is by chemical functionalization, because XG has a large amount of hydroxyl (−OH) and free carboxyl groups (COO−), such as methacrylation,^13^ copolymerization,^14^ and amidation reactions.^15^ However, these reactions face several drawbacks due to the high XG viscosity, including the low diffusion and reduced reactivity of the reaction agent, difficulty in separating agents by centrifugation, and complications in purification because capillaries become clogged, followed by a low dialysis efficiency. Hence, they are often long processes with an overall low efficiency. A few studies that pursued this route employed XG blended with other polymers.^13,14^

Lastly, the use of divalent and trivalent ions for the ionic crosslinking of XG has been reported.^3,15–17^ Yet, most studies have blended XG with other materials, for example, XG–alginate,^3^ where Ca^2+^ was explored, or mixing XG–carboxymethyl cellulose and hyaluronic acid,^16^ where Fe^3+^ was employed. DOPA-conjugated XG was also established, whereas H_2_O_2_ and Fe^3+^ were used as crosslinkers.^15^ Although these studies bring relevant knowledge to our aim, the sole XG crosslinking effect by these ions was hindered by the other polymer components. Kang et al. were among the first to report the use of trivalent ions (Fe^3+^) for solely XG, as a sensor to detect oxidizing and reducing agents coupled to lactate.^17^ The process was based on a long 18 h reversible sol–gel process using molding techniques. However, cell toxicity effects were not investigated.^17^ Similarly, Fe^3+^ was also employed for crosslinking alginate hydrogels,^18^ whereas cell toxicity effects were also not explored.

Besides these limitations, XG has shown great potential in tissue engineering applications and drug delivery systems, as extensively revised elsewhere.^7,19^ Yet, XG has been solely employed via injection^20–22^ thus not necessarily requiring crosslinking or relying on the presence and crosslinking efficiency of other materials,^3,13,16,23^ or both.^15^ Interesting in vivo studies using XG injections were already reported, which reduced cartilage degradation and slowed cell death and inflammation in osteoarthritis rabbit and rat models.^20–22^ Hence, we envision a novel crosslinking strategy for XG that enables its sole use in biomedical engineering applications, rather than using it solely as a thickening agent for bioink design, while allowing its processability via additive manufacturing.

We first present an ionic crosslinking strategy to XG that allows the manipulation of its physicochemical and mechanical properties, without using toxic agents or requiring prior chemical modification, to obtain a stable, biocompatible, and inert hydrogel system (XG–Fe^3+^) for culturing with cells. We introduce a new biological functionality by adding human protein-derived methacryloyl platelet lysates (hPLMA), thus transforming it from an inert into a bioactive hydrogel (XG–Fe^3+^–PLMA). Platelet lysates recently arose as a source of highly biocompatible materials that can drive tissue regeneration due to their innate cell-recruiting and pro-regenerative capacities.^24^ The possibility of crosslinking them using light opens new possibilities for developing human-derived matrices for 3D cell culture, either for therapeutic purposes or drug screening applications.^25–29^ Hence, we aimed to provide a versatile XG–Fe^3+^ hydrogel platform to enable the sole use of XG either as an inert hydrogel for drug delivery purposes or as a bioactive hydrogel for biomedical engineering applications. A screening among five ions and a large window of concentrations was performed, followed by extensive rheological and mechanical characterization. XG hydrogels were 3D printed in complex patterns that achieved a centimeter scale. Stability and swelling effects in the culture conditions of the printed scaffolds were investigated over two months. The performance of XG–Fe^3+^ hydrogels as a carrier for cells (hMSCs) and bioactive molecules with low stability in aqueous solutions (hPLMA) was demonstrated with high cell viability and metabolic activity. Finally, two validations of the XG–Fe^3+^ hydrogel in more complex scenarios were pursued. First, we encapsulated magnetic nanoparticles (MNPs) in XG–Fe^3+^ hydrogels to analyze their response to external stimuli and expand their carrier role to a delivery role as a remotely stimulated scaffold. Thus, magnetically responsive XG–Fe^3+^–MNP hydrogels were obtained. Second, hybrid scaffolds coupled with a gold-standard thermoplastic material (polycaprolactone, PCL) were developed. Thus, XG–PLMA–PCL hybrid scaffolds were obtained by using a multiprinting approach that combines 3D printing and melt electrowriting (MEW) in a single piece of equipment. Ultimately, due to the current attention on developing tailored scaffolds for complex tissue engineering applications, we foresee the potential of the XG–Fe^3+^–PLMA system, given its remarkable versatility as a natural, mechanically tunable, and bioactive hydrogel. Moreover, whether used in pure or hybrid scaffolds with PCL, encapsulating magnetic particles, or not, we foresee broad applications for biomedical engineering purposes. This set of capabilities is rarely observed in other non-modified natural polysaccharides.

## Results

### Screening, Mechanical and Chemical Characterization of Several Bi and Trivalent Ions for Ionic Crosslinking of Xanthan Gum

Although XG performs significantly better than other polysaccharides available for biomedical engineering applications, its low mechanical performance without crosslinking hinders its sole application. Because XG has many hydroxyl groups (−OH) and free carboxyl groups (COO−), the studies that did not combine XG with another material performed chemical functionalization utilizing these groups (Figure 1B). Herein, our goal was to take a third route, manipulating and optimizing the mechanical and physicochemical properties of XG solely without prior chemical modification (Figure 1C). Hence, we first assessed the in situ ionic crosslinking effects of different cations, e.g., Ca^2+^, Mg^2+^, Fe^2+^, Al^3+^, and Fe^3+^, on XG hydrogels by a rheological assessment using a rheometer equipped with a homemade cup-shaped accessory.^30^ These in situ crosslinking experiments allowed us to precisely monitor the crosslinking efficiency and stiffness. The initial *G*′ of the XG hydrogels was assessed (0.9 ± 0.016 kPa). Then, after adding 50 mM of salt solutions (Ca^2+^, Mg^2+^, Fe^2+^, Al^3+^, and Fe^3+^) for ionic crosslinking for 15 min, the storage modulus *G*′ (Figure 1D), time sweeps of the storage modulus *G*′ (Figure 1E), and loss modulus of *G*″ (Figure S1) were assessed. Because these experiments were based on the ion effect, Milli-Q water was used as a hydrogel solvent to avoid ion precipitation and eliminate undesirable crosslinking effects of other ions present in salt-based buffers, e.g., PBS. XG hydrogels showed a great increase in storage modulus in the presence of trivalent cations (Al^3+^ and Fe^3+^), while divalent cations (Ca^2+^, Mg^2+^, and Fe^2+^) did not demonstrate any significant contribution to the stiffness, indicating that divalent cations cannot crosslink XG (Figure 1D–G).

Interestingly, there was a high affinity of XG for Fe^3+^ ions rather than for Al^3+^. XG hydrogels crosslinked with 50 mM Fe^3+^ for 15 min were 3 times stiffer than XG crosslinked with Al^3+^ (*G*′ = 93 ± 3.5 kPa and *G*′ = 29 ± 0.9 kPa, respectively, Figure 1F). The loss modulus was also assessed (Figure S2). These measurements were performed by using a rheometer with a 1000 μm geometry gap. To further investigate this affinity, we analyzed different concentrations of Al^3+^ ranging from 25 to 100 mM, which resulted in no significant stiffness improvement even at the highest concentration (Figure S3). Furthermore, we identified that the crosslinking effect of Al^3+^ on XG was already saturated when using 25 mM of Al^3+^ (Figure S3). Then, due to the high affinity of XG polymer chains for Fe^3+^ using 50 mM, we investigated XG stiffness when using lower Fe^3+^ concentrations (5, 10, 25, and 50 mM). To achieve the required accuracy of rheological assessment when using low Fe^3+^ concentrations, we employed a low geometry gap of 500 μm. Contrasting with Al^3+^ behavior, the stiffness of XG hydrogels was increased with Fe^3+^ in solution after 15 min (4.2 ± 0.03 kPa for 5 mM, 26.8 ± 0.8 kPa for 10 mM, 90 ± 3 kPa for 25 mM, and 143 ± 9 kPa for 50 mM, Figure 1G, open symbols). The loss modulus *G*″ was also assessed (Figure S4). Finally, lower concentrations than 5 mM Fe^3+^ (0.1, 0.25, 0.5, and 1 mM) were assessed (Figure S5) and all of them did not show any effect on stiffness. Hence, Fe^3+^ is the best crosslinking agent for XG among the five cations herein explored when using concentrations from 0.1 up to 50 mM.

Once the Fe^3+^ crosslinking effect on XG was elucidated in Milli-Q, we investigated the effect of dissolving Fe^3+^ in PBS buffer (which intrinsically contains divalent ions) to mimic the cellular microenvironment for tissue engineering applications (Figure 1G, filled symbols). For 50 mM Fe^3+^, the stiffness of XG hydrogels was similar in both PBS and water (140 ± 34 vs 143 ± 9 kPa, respectively). Curiously, a much lower stiffness was assessed using PBS rather than water for 25 mM Fe^3+^(40 ± 9.4 kPa vs 90 ± 3 kPa, respectively), and minimal crosslinking effects were observed for 10 and 5 mM of Fe^3+^ when PBS was used as a solvent (1.9 ± 0.6 kPa and 1.7 ± 0.4 kPa, respectively). Moreover, salt precipitation was observed at low Fe^3+^ concentrations (10 and 5 mM dissolved in PBS). Thus, we investigated the effect of pH on the XG–Fe^3+^ environments.

To analyze the pH effect (Figure S6) on the XG–Fe^3+^ microenvironment, we first assessed the pH values of several FeCl_3_ aqueous solutions (100 μM: pH = 7.5; 250 μM: pH = 7.4; 500 μM: pH = 7.3; 1 mM: pH = 7.3; 25 mM: pH = 2.1; and 50 mM: pH = 2.1) and the acidic level increased with the FeCl_3_ concentration. Then, we increased the pH of the highest FeCl_3_ solution (50 mM) by dropping NaOH (1 M) until neutral (6.2–7.2) and basic values (11.8–12.2) in both PBS aqueous and XG hydrogel solutions. When working on acidic pH (1.7–2.2) with 25 and 50 FeCl_3_ mM, the hydrogels were homogeneously crosslinked with a clean dark yellow visual appearance. When working on neutral (6.2–7.2) and basic values (11.8–12.2) with 25 and 50 mM FeCl_3_, XG hydrogels were not crosslinked, and high brown precipitation was observed. Interestingly, when working with 1 mM FeCl_3_, no precipitation and no crosslinking were observed on any pH scale. Hence, our results show that when adding NaOH, the following reaction occurred: FeCl_3_ + NaOH → Fe­(OH)_3_ + NaCl, resulting in the precipitation of ferric oxide (Fe­(OH)_3_). Ferric oxide has very low solubility in water-like environments, and although most metals precipitate in water from pH 6–9, ferric iron begins to precipitate at pH = 2.5.^31^ Because the most dominant iron form in neutral and basic pH is Fe^2+^ (ferrous),^31^ we assessed that both in PBS aqueous and XG hydrogel solutions, ferric iron (Fe^3+^) was reduced to ferrous iron (Fe^2+^), which disrupted the crosslinking effect of XG–Fe^3+^ and precipitated as ferric oxide Fe­(OH)_3_. Hence, an acidic environment is necessary to obtain the strong crosslinking effect of XG using Fe^3+^ and the typical yellow-brown color change helps in identifying when the crosslinking effect is broken. Next, we investigated XG stability over temperature variations using digital scanning calorimetry (Figure S7). XG was very stable from −90 to 100 °C (101.72 °C as the peak temperature) before starting its thermal degradation. Finally, we also investigated oxygen concentrations in XG–Fe^3+^ for 50 h and compared them to the classical alginate–Ca^2+^ system, as well as alginate–Fe^3+^ and PBS as a control. Similar results were observed, XG–Fe^3+^ was maintained between 17 and 18% of the oxygen concentration over 50 h (same as PBS as the control), while both alginate groups were maintained between 16 and 17% (Figure S8). So, by only changing the Fe^3+^ concentration in acidic pH, we could easily tailor the mechanical properties of XG to achieve values from 0.9 ± 0.016 kPa to 143 ± 9 kPa, demonstrating the high versatility of this hydrogel system for several applications.

### 3D Printing, Stability, and Morphological Evaluation of XG Hydrogels

The crosslinking effect of XG–Fe^3+^ 50 mM led to stable and robust 3D-printed hydrogel scaffolds. Scaffolds could sustain their weight (Figure 2A), while without crosslinking, they could not (Figure 2B). XG 7 wt % demonstrated an excellent printing performance. Scaffolds with 25 layers and 1.5 cm were obtained without collapsing (Figure 2C), which is remarkable for natural hydrogels without prior modification. Moreover, many standard and complex printing patterns were easily manufactured with high fidelity (Figure 2D), including the Gosper curve (Figure 2E), which is challenging for standard hydrogels due to its large number of turns while continuously extruding. Scaffolds (*N* = 8) were produced in a fast (2.5 min, 20 s per scaffold) and reproducible process (Figure S9) and attached very well to standard non-treated well plates (Figure 2F). The smallest filaments obtained (414 μm in diameter) using XG 7 wt % were manufactured at high speed (70 mm/s) and were intact and stable (Figure S10). Then, we moved to investigate the stability under culture conditions of XG–Fe^3+^ scaffolds. Remarkably, XG–Fe^3+^ scaffolds remained stable for 60 days, with the maintenance of printing architecture (Figure 2G). However, an intense swelling was observed. Scaffolds increased around 162% in the overall area after 30 days and 181% after 60 days (Figure 2H, *N* = 5, purple and green lines are helpful for a visual comparison). When calculating the ratio of the increased area of XG–Fe^3+^ scaffolds (Figure 2I, *N* = 5), we found that the increase of 38% occurred from day 1 to day 7. From day 7 to 30 days, an increase of 13% was observed, and from day 30 to 60 days, 18%. Hence, a rapid swelling occurred in the first 7 days in culture, followed by a smooth increasing trend over two months. Nevertheless, the pores were maintained open (Figure 2J) and showed a consistent size over the whole period (day 0 after crosslinking until day 60), ranging from 206 to 246 μm in average (Figure 2K).

**Figure 2:**
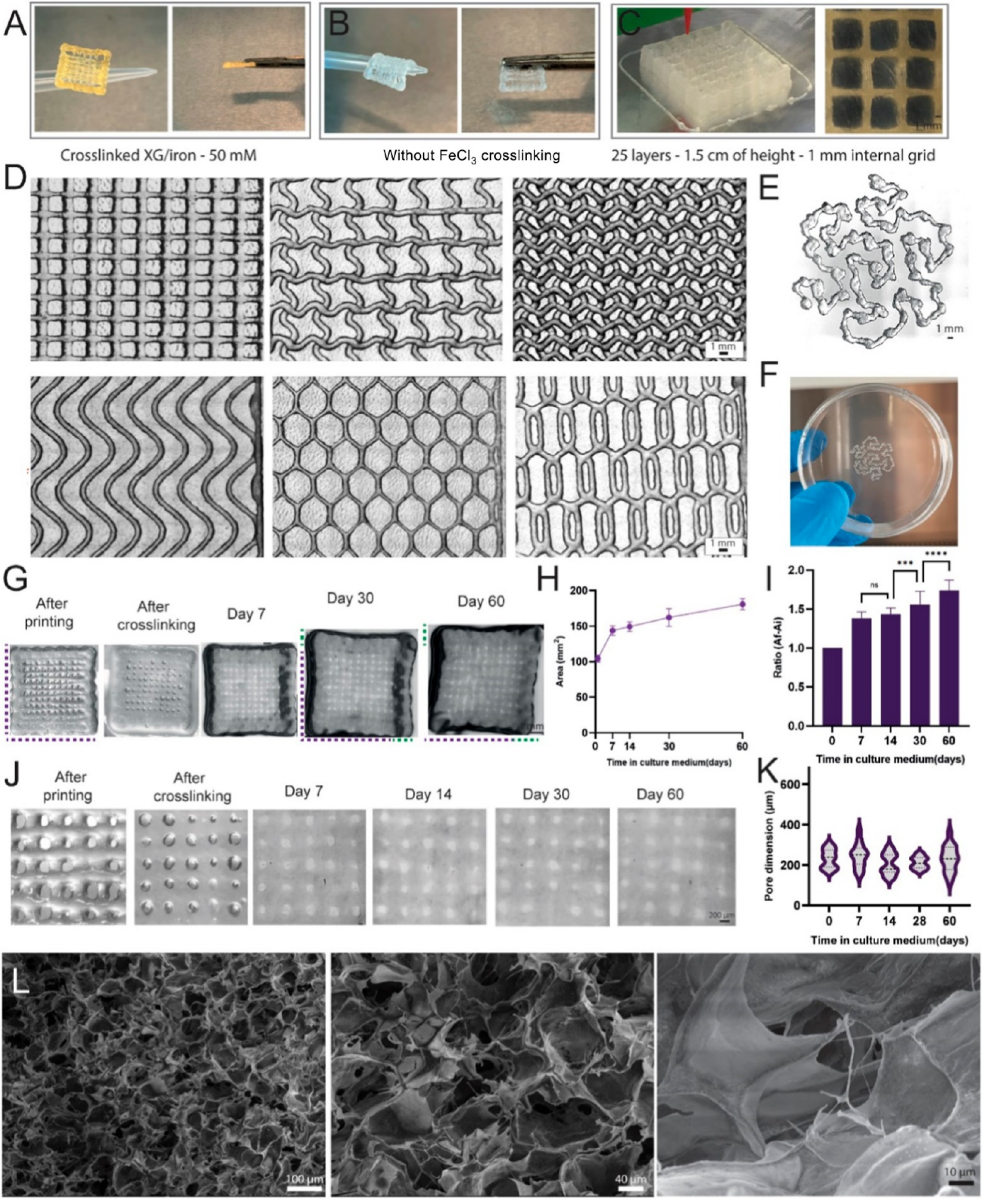
3D Printing, stability, and morphological evaluation of XG–Fe^3+^ hydrogels. (A) Visual aspects of the strong crosslinking effect of XG–Fe^3+^ 50 mM on 3D printed scaffolds and (B) in the absence of crosslinking. (C) Scaffolds with up to 25 layers and 1.5 cm were obtained without collapsing. (D) Several standard and complex printing patterns were easily manufactured by an extrusion-pressure system with high fidelity, including the challenging (E) Gosper curve, characterized by its large number of turns while continuously extruding, and (F) scaffolds easily attached to nontreated well plates. (G) XG–Fe^3+^ scaffolds remained stable for 60 days, maintaining a printing architecture. (H) Increased area and (I) ratio of XG–Fe^3+^ scaffolds over 60 days due to the swelling effect. (J) Maintenance of open pores with (K) a consistent size over 60 days. (L) Scanning electron microscopy (SEM) micrographs of XG 7% evidencing details on the surface structures.

Scanning electron microscopy (SEM) analysis showed the surface micromorphology of the XG hydrogels (Figure 2L). As expected, a dense hydrogel structure was observed but with a heterogeneous microporous surface (43 ± 23 μm), which benefits cell interactions and facilitates nutrient exchange. Hence, XG–Fe^3+^ hydrogels demonstrated exceptional printability for printing complex patterns and outstanding stability in cell culture environments, indicating high potential as a carrier for cells and proteins with low stability in aqueous solutions.

### XG–Fe^3+^ Hydrogels as a Carrier of Cells and Bioactive Molecules with Low Stability in Aqueous Solutions

To investigate the performance of XG–Fe^3+^ hydrogels as a carrier for cells and proteins with low stability in aqueous solutions, we first examined cell behavior using human mesenchymal stem cells (hMSCs). Since an acidic environment is necessary to trigger the crosslinking effect of XG using Fe^3+^, encapsulating cells for bioprinting was not possible because cell metabolic activity decreased with an increasing Fe^3+^ concentration in the solution (Figure S11). However, this fact was caused exclusively by the acidic pH because when we seeded hMSC on XG–Fe^3+^ scaffolds, previously prepared and washed in water to remove unbound iron in excess. The hMSC metabolic activity at day 7 was the same whether using 25 or 50 mM Fe^3+^ or without any Fe^3+^ addition (Figure 3A).

**Figure 3:**
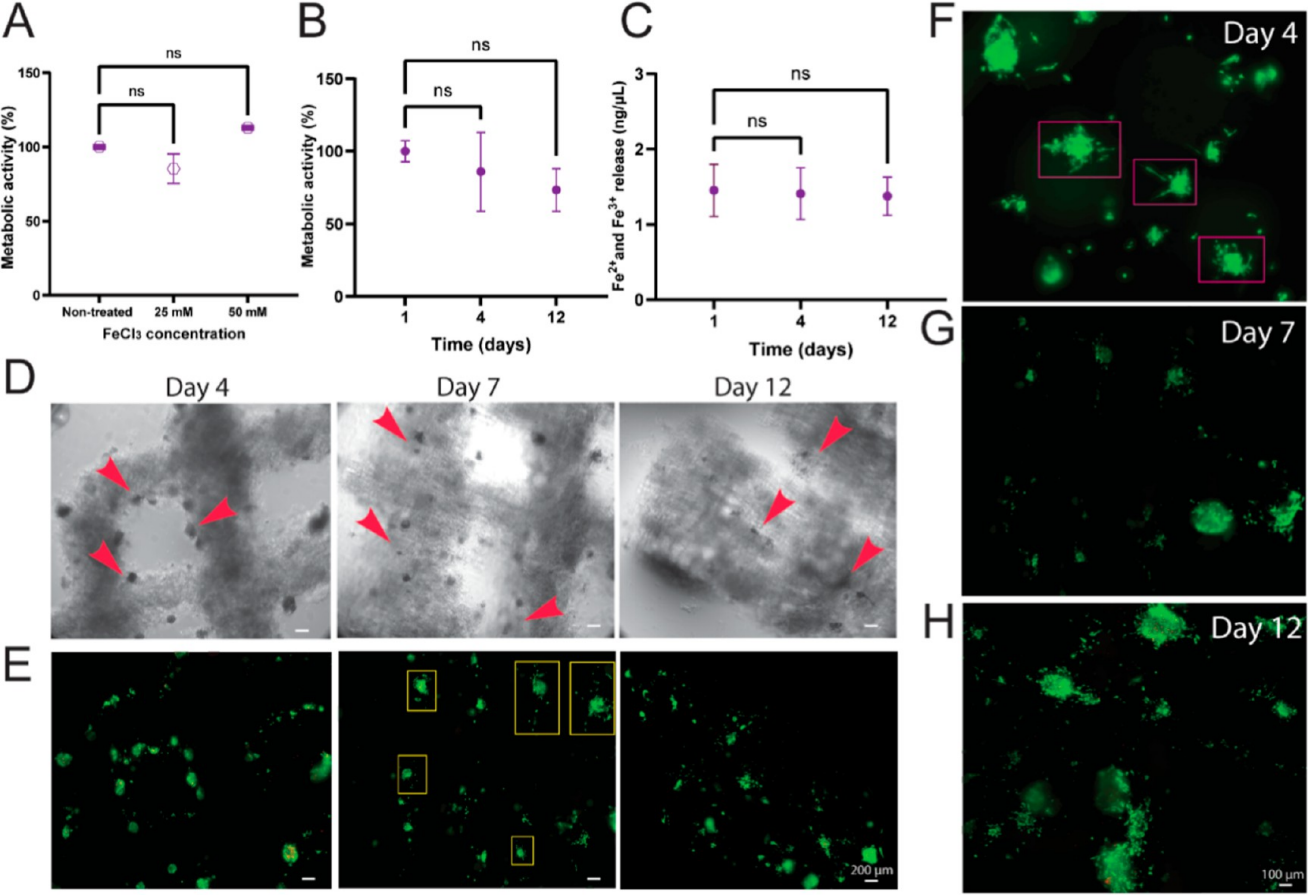
XG–Fe^3+^ hydrogels as a carrier of cells. (A) Metabolic activity of hMSCs at day 7 on XG7 scaffolds crosslinked using 25 or 50 mM Fe^3+^ and in the absence of Fe^3+^ and (B) metabolic activity of hMSCs over 12 days of culture on scaffolds crosslinked using 50 mM Fe^3+^. (C) Smooth and steady release of Fe^3+^ and Fe^2+^ over 12 days of culture using 50 mM Fe^3+^. One-way ANOVA tests were performed and the results indicated non-significant differences for *P* ≥ 0.05 for all graphs (ns). (D) Spreading of hMSCs within the XG scaffolds for 12 days. From cell seeding to day 4, cells were aggregated in small spheroids which were reducing in size over time (red arrows). (E) High cell viability after 4, 7, and 12 days in XG–Fe^3+^ scaffolds using 50 mM Fe^3+^. (F) Details on the structure of cell aggregates that started to spread on the surface of XG (pink squares). (G) At day 7, the size of hMSC aggregates was reduced and cell infiltration toward the XG scaffolds was observed. (H) At day 12, hMSC cells were spread all over the whole scaffold, including surface and internal areas.

We also assessed that metabolic activity was well maintained over 12 days of cell culture, with no statistically significant difference when using 50 mM Fe^3+^ (Figure 3B). Next, we evaluated the release of Fe^3+^ and Fe^2+^ over this time frame (12 days), and a smooth and steady release was observed, without any harmful burst effect (Figure 3C). Then, we moved to analyze cell viability, position, and spreading of hMSCs within the XG scaffolds for 12 days (Figure 3D). High cell viabilities after 4, 7, and 12 days in XG–Fe^3+^ scaffolds using 50 mM Fe^3+^ were observed (Figure 3E). Interestingly, from cell seeding to day 4, the cells were aggregated in small spheroids (133 ± 26 μm in diameter, *N* = 10) on the surface of XG filaments with 679 ± 89 μm in diameter (Figure 3D red arrows, day 4 and Figure 3E, day 4). At day 7, the size of these aggregates was reduced due to their partial disaggregation, followed by cell spreading and infiltration toward the XG scaffolds (Figure 3D red arrows, day 7 and Figure 3E, yellow squares, day 7). At day 12, hMSCs were spread over the surface and inside the whole scaffolds (Figure 3E, day 12). More details on the aggregate structure, cell detachment from the aggregate (pink squares), and spreading can be seen in replicate samples with a higher magnification (Figure 3F–H). Hence, using XG–Fe^3+^ scaffolds up to 50 mM Fe^3+^ is safe for hMSC seeding and culture and allows for hMSC infiltration and spreading from the surface into the hydrogel after 12 days.

Next, we investigated the performance of XG–Fe^3+^ hydrogels as carriers of bioactive molecules essential for the growth and function of hMSCs, which typically exhibit low stability in aqueous environments. For the source of bioactive factors, we selected human platelet lysates (PL) because they have recently arisen as a source of highly biocompatible materials that can drive tissue regeneration due to their innate cell-recruiting and pro-regenerative capacities.^24^ Nevertheless, their sole use as scaffolds is severely compromised due to their intrinsic low viscosity and unstable character.^28,29^ The PLs were functionalized by the addition of methacrylic groups to obtain photopolymerizable hydrogels as previously described^28,29^ and XG–hPLMA hydrogels were obtained by mixing XG 7% with 10, 15, 20, and 30% of hPLMA (XG7–PLMA10, XG7–PLMA15, XG7–PLMA20, and XG7–PLMA30, respectively).

First, due to the low viscous character of hPLMA (viscosity values close to water, 10^–3^ Pa·s, as a Newtonian fluid),^29,32^ these four formulations were assessed regarding printability. When assessing printing performance in scaffolds up to 8 layers while decreasing the pitch (Figures S12 and S13), we concluded that there was poor printing fidelity and resolution for XG7–PLMA20 and XG7–PLMA30. As expected, hPLMA harmed printability when employed in high concentrations (XG7–PLMA30 and XG7–PLMA20), while lower concentrations (XG7–PLMA10 and XG7–PLMA15) were able to demonstrate satisfactory printing fidelity (Figures S12 and S13). Thus, we assessed the viscosity of both solutions with lower hPLMA fractions (XG7–PLMA10 and XG7–PLMA15) and compared them with pure XG7 and hPLMA alone (Figure 4A). hPLMA has a much lower viscosity than pure XG7, XG7–PLMA10, and XG7–PLMA15 blends, which presented similar viscosities. Next, we focused on 3D printing two and eight-layer scaffolds. The 3D structure and open pores were maintained within both blends (Figure 4B) with stability after eight layers being deposited (Figure 4C). Because XG7–PLMA15 filament diameters were larger (1359 ± 34 μm) than XG7–PLMA10 (1110 ± 26 μm), we selected XG7–PLMA10 to proceed to stability and cell culture studies.

**Figure 4:**
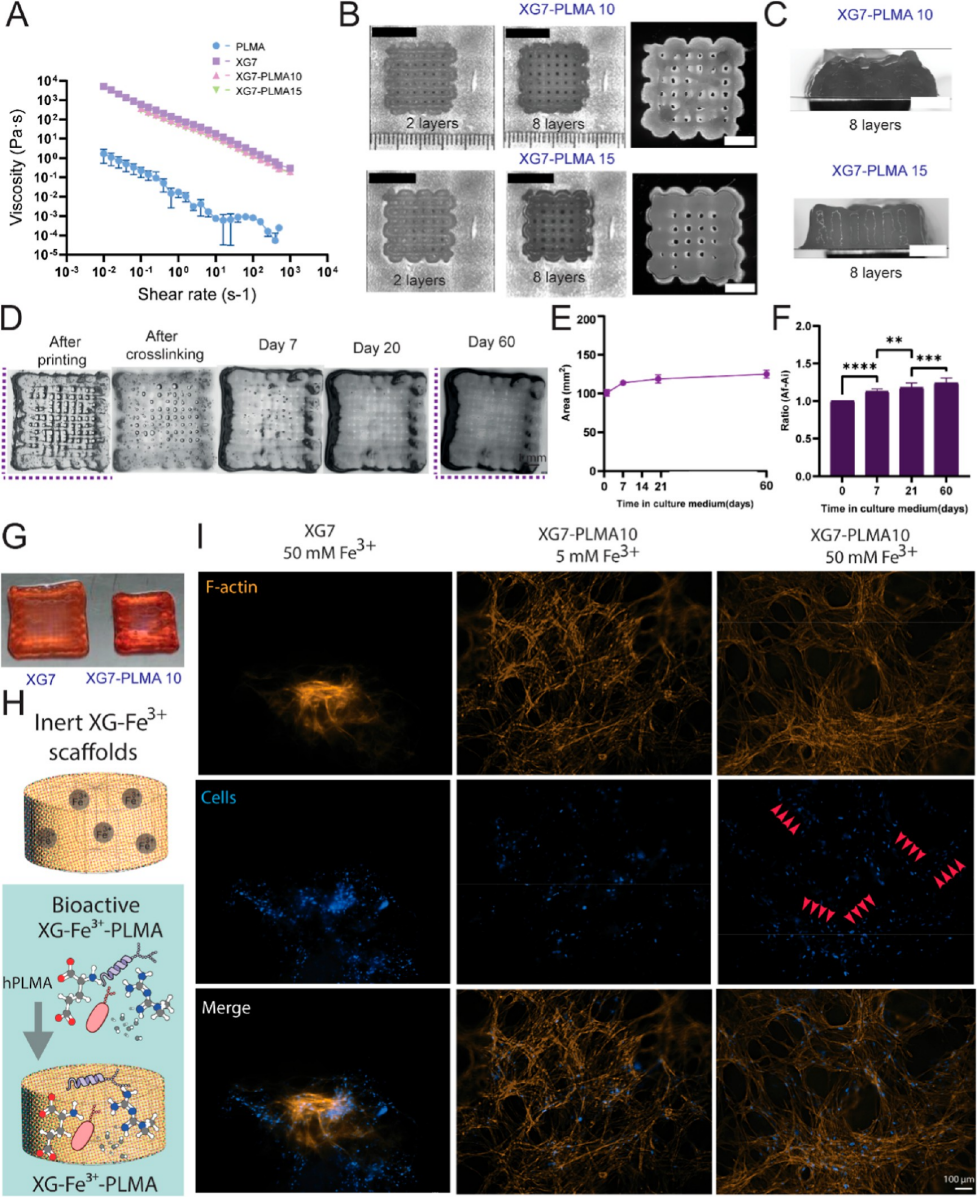
XG–Fe^3+^ hydrogels as a carrier of bioactive molecules (hPLMA) with low stability in aqueous solutions. (A) Viscosity of pure XG 7% (w/v) hydrogel when compared to blending it with hPLMA (XG7PLMA10) and pure hPLMA. (B) Manufacturing scaffolds with two and eight layers using XG7–PLMA10 and XG7–PLMA15 blends. Scale bar is 5 mm (C) coalescence effect above manufacturing eight-layer scaffolds using XG7–PLMA10 and XG7–PLMA15 blends. Scale bar is 5 mm. (D) XG–Fe^3+^–PLMA scaffolds are stable for 60 days, ionically and photocrosslinked, with the maintenance of open pores and printing architecture. (E) Scaffold area and (F) ratio of XG–Fe^3+^–PLMA scaffolds over 60 days. (G) Swelling effect when using the single crosslinking strategy on XG–Fe^3+^ and double crosslinking strategy on XG–Fe^3+^–PLMA hydrogels. (H) Schematic illustration of the inert XG–Fe^3+^ and the bioactive XG–Fe^3+^–PLMA10 systems. (I) F-actin (orange) and cell nuclei (blue) staining comparing the behavior of hMSCs after 7 days in culture when using the single crosslinking strategy on XG–Fe^3+^ hydrogels and double crosslinking strategy on XG–Fe^3+^–PLMA scaffolds varying Fe^3+^ from 5 to 50 mM.

In contrast to XG–Fe^3+^ scaffolds, where an intense swelling effect was noted, resulting in an increase of 81% in the their area after 60 days, XG7–PLMA10 scaffolds demonstrated only a 25% increase in area over 60 days (Figure 4D,E). When calculating the ratio of increased area of XG7–PLMA10 scaffolds (Figure 4F, *N* = 5), we observed an increase of 13% from day 1 to 7, and 7% from day 21 to 60. Hence, a slow and steady swelling occurred over the whole 60-day period when using double crosslinked XG7–PLMA10, contrasting with the rapid increase in ionically crosslinked XG–Fe^3+^ scaffolds over the first weeks in culture. Similarly to XG–Fe^3+^ scaffolds (Figure 2G), open pores and the printing architecture were also maintained over 60 days (Figure 4D). Hence, our results have shown that the addition of hPLMA and a double crosslinking approach controlled the swelling effect of XG (Figure 4G). This is a great improvement as the intense swelling is often cited as a major drawback of XG.

The behavior of hMSCs when comparing the inert XG–Fe^3+^ with the bioactive XG–Fe^3+^–PLMA10 (Figure 4H) was assessed using XG7–PLMA10 scaffolds and 5 and 50 mM Fe^3+^ (Figure 4I). Using XG7–PLMA10, cells were spread and attached to binding hPLMA points, forming organized cell structures in the hydrogel with a considerably increased production of F-actin when hPLMA was included (50 mM). Interestingly, when using 5 mM Fe^3+^, actin filaments and cells were spread diffusely and randomly, whereas with 50 mM Fe^3+^, they followed a more organized structure with lined patterns (Figure 4I, red arrows). Hence, the addition of PLMA turned the inert XG scaffolds into a bioactive system. XG7–PLMA10 hydrogels are both mechanically and biologically relevant for tissue engineering applications, allowing for the 3D printing of complex patterns with high fidelity and functionality.

Next, we investigated the effects of crosslinking strategies on mechanical properties, comparing the use of a single crosslinking on XG–Fe^3+^ to the double crosslinking on XG–Fe^3+^–PLMA hydrogels (Figure 5A). By comparing XG7 without crosslinking, with XG7–Fe^3+^ 50 mM, and blended with PLMA to obtain the bioactive XG7–Fe^3+^–PLMA10, we could identify how each component addition or crosslinking methodology contributed to the overall mechanical properties.

**Figure 5:**
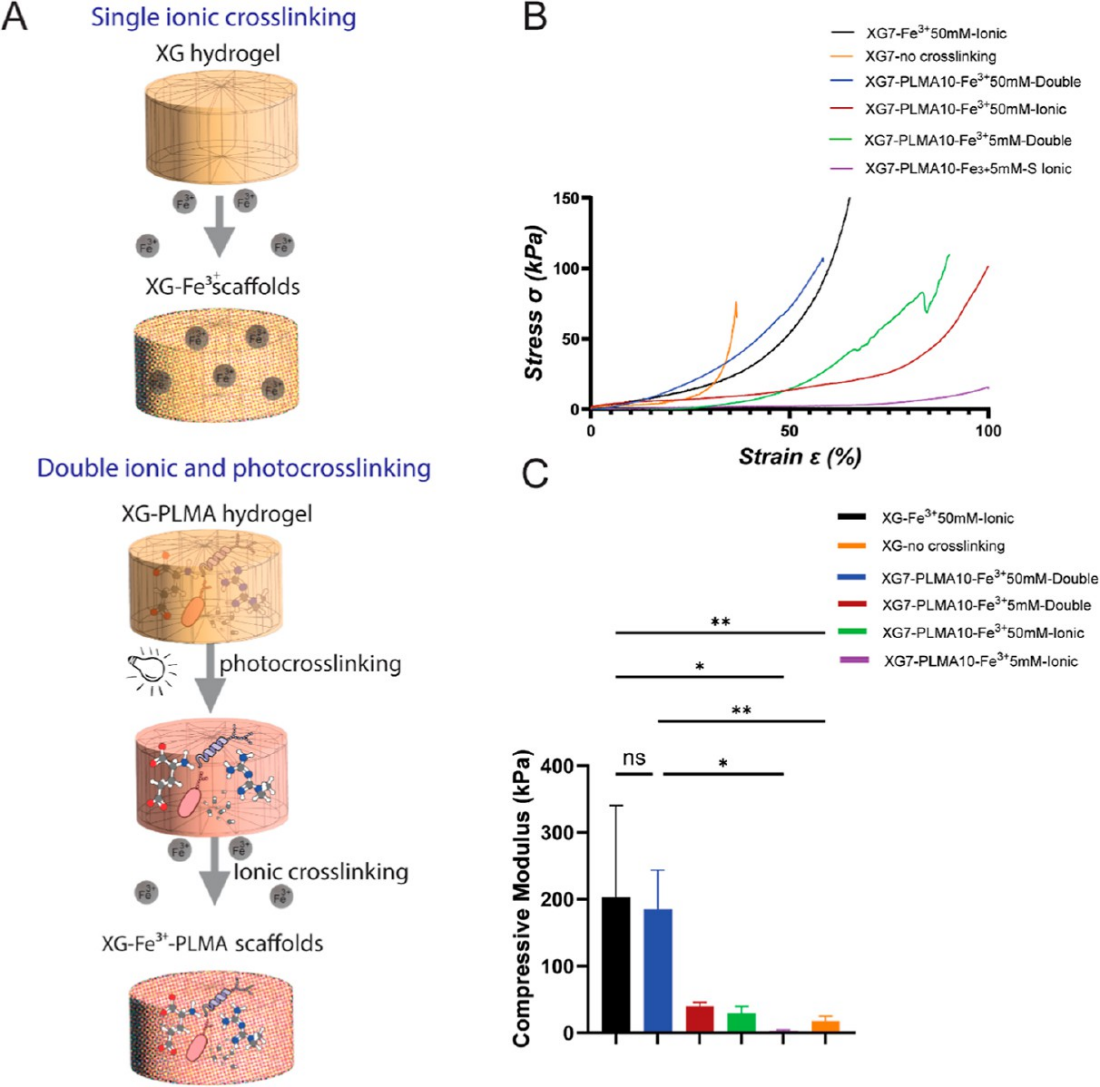
Mechanical properties of XG7–Fe^3+^ and XG7–Fe^3+^PLMA throughout all hydrogel conditions when employing the (A) single ionic crosslinking strategy using Fe^3+^ ions and its combination with photocrosslinking of methacrylate groups in hPLMA to obtain the double crosslinked XG7–Fe^3+^PLMA scaffolds. (B) Stress/strain curves and (C) compressive modulus within the linear region of the stress/strain curves at 30–35% deformation. Throughout all hydrogel conditions (significant differences *, *p* ≤ 0.05; **p ≤ 0.005, ns for not-significant differences, using a one-way ANOVA test).

Stress/strain curves were used to display the accumulation of forces throughout all hydrogel conditions (Figure 5B) and from which the compressive modulus was calculated within the linear region of the stress/strain curves at 30–35% deformation (Figure 5C). When comparing the XG–Fe^3+^ 50 mM hydrogel (black) with XG not crosslinked (orange), a nearly 10-fold higher resistance to strain is noticed in the ionically crosslinked XG–Fe^3+^ 50 mM hydrogel (202.7 ± 137.8 kPa), while non-crosslinked XG demonstrated little compressive strength (18.0 ± 7.0 kPa). These compressive moduli corroborate the stiffness differences obtained by a rheological assessment in the presence or absence of iron (Figure 1G). Interestingly, when using 50 mM Fe^3+^ for both single ionically crosslinked XG7–Fe^3+^ (black) and double crosslinked XG7–Fe^3+^50–PLMA10 (blue), the greater mechanical properties with an increased compressive modulus were obtained among all groups, but with no considerable differences among these two groups (202.7 ± 137.8 vs 185.5 ± 57.9 kPa, respectively, ns for not-significant). On the other hand, when comparing the same hydrogels but using a lower iron amount (5 mM), much inferior mechanical performances were observed in both single (purple) and double crosslinking strategies (green) (3.2 ± 1.3 vs 39.9 ± 5.6 kPa, respectively). The compressive modulus of un-crosslinked XG7 (orange) was higher (18.0 ± 7.0 kPa) than XG7–PLMA10 ionically crosslinked using the lower 5 mM Fe^3+^ amounts (pink), which demonstrated no compression strength (3.2 ± 1.3 kPa), corroborating with their very low stiffness (4.2 ± 0.03 kPa) in the rheological assessment (Figure 1G). This indicates that the addition of PLMA initially lowers the baseline stiffness, affecting the overall performance of the XG7 precursor and requiring a higher Fe^3+^ concentration (green) or the double crosslinking approach (red) to regain the original XG7 stiffness levels. Hence, we overall identified that the Fe^3+^ concentration had a much more pronounced effect on XG mechanical properties than the simultaneous use of two combined ionic and photocrosslinking strategies.

After proving that XG–Fe^3+^ can be mechanically tunable while turned into a bioactive hydrogel system, we tested XG–Fe^3+^ system for two other proof-of-concept applications. First, we analyzed whether XG–Fe^3+^ responds to external stimuli to expand its carrier role of cells and bioactive compounds to a delivery role using magnetic nanoparticles. Second, we analyzed the suitability of XG–Fe^3+^–PLMA for enabling the convergence of 3D printing and melt electrowriting (MEW). For this purpose, polycaprolactone (PCL) was included to obtain hybrid scaffolds (XG–Fe^3+^–PLMA–PCL), thereby leveraging the advantages of combining natural and synthetic materials.

### XG–Fe^3+^ Hydrogels as a Printable Delivery System That Can Respond to Magnetic Actuation

Two forms of iron nanoparticles were initially explored: hematite and magnetic particles (MNPs). After screening hydrogel formulations ranging from 2 to 7% XG (w/v) within 2.5% of hematite or MNPs by the inversion tube technique (Figure S14), XG7–Fe^3+^–hematite and XG7–Fe^3+^–MNPs were selected and manufactured. XG7 printability was not affected by the encapsulation of nanoparticles (average filament sizes of four-layered scaffolds were 616 ± 85, 562 ± 68, and 589 ± 45 μm for XG7–Fe^3+^, XG7–Fe^3+^–MNPs, and XG7–Fe^3+^–hematite, respectively, when employing the same printing parameters). Satisfactory printing performance, without filament rupture, was observed even at a high printing speed of 70 mm/s (Figure 6A). Both hematite and MNPs scaffolds were homogeneous and stable over time after Fe^3+^ 50 mM crosslinking. Because hematite is a nonmagnetically responsive iron oxide, we proceeded to use only MNPs to investigate magnetic actuation. Interestingly, when mixing single and dispersed MNPs (diameters ranging from 100 to 200 nm, assessed by TEM microscopy, Figure S15) in XG7 hydrogels, the particles clustered into MNP aggregates in certain areas of the printed scaffolds, mostly due to the high thickening effect of XG (Figure S16). When first MNPs were dissolved in the solvent with further XG addition during constant agitation, reproducible XG–Fe^3+^–MNP scaffolds were obtained with a homogeneous distribution of MNPs in the overall scaffold, thus providing an opportunity to control remote actuation spatially (Figure 6B). Hence, two opportunities were identified, e.g., the application of a homogeneous MNP distribution when envisioning a holistic scaffold magnetic response, or concentrated MNP distribution to apply the magnetic effect in specific scaffold areas while maintaining it to be nonresponsive in other areas.

**Figure 6:**
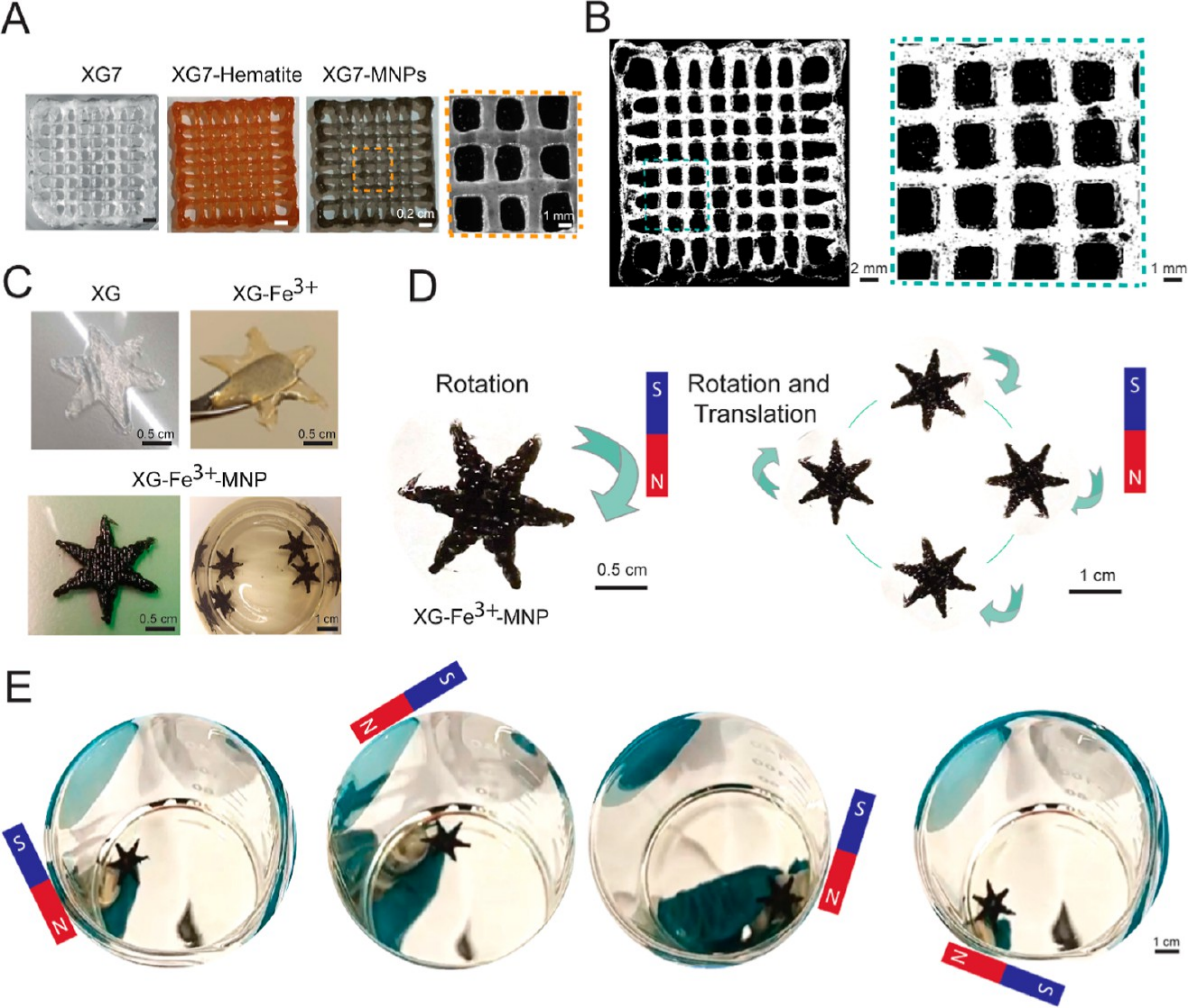
Magnetically responsive XG–Fe^3+^–MNP scaffolds. (A) Manufacturing of XG–Fe^3+^–hematite and XG–Fe^3+^–MNP scaffolds. (B) Homogenous distribution of MNPs (black dots) in XG–Fe^3+^ hydrogels obtained by an optimized hydrogel preparation protocol. (C) Manufacturing star-shaped scaffolds with a homogeneous distribution of MNPs in XG–Fe^3+^ hydrogels to evaluate the scaffold magnetic response. (D) MNPs embedded in XG 25 mM Fe^3+^ hydrogels followed the permanent magnet stimulation to perform rotation and translation. (E) Rotation and translation movements were easily performed with trajectory speed dictated by the magnet orientation.

To evaluate the magnetic response in the XG–Fe^3+^–MNPs scaffolds, we printed scaffolds with a star shape. Concentrations of 10 and 25 mM Fe^3+^ proved ideal for crosslinking while allowing the scaffolds to be easily manipulated as a whole using a permanent magnet (Figure 6C). When using 5 mM Fe^3+^, the hydrogels were soft and could not be used for assessing magnetic actuation, while 50 mM Fe^3+^ was too stiff. When positioning a permanent magnet, the MNPs embedded in the XG 25 mM Fe^3+^ followed the magnetic stimulation with a speed dependent on the actuation speed. Stars performed rotation and translation movements over a circular trajectory (Figure 6D,E and Video S1). Therefore, even with the intense thickening effect of XG 7%, the stiff hydrogel character due to a high XG–Fe^3+^ crosslinking affinity, and the presence of microparticles (MNP), the XG–Fe^3+^–MNP scaffolds proved to be magnetically responsive. Hence, XG–Fe^3+^–MNP scaffolds can be remotely stimulated for interesting biomedical engineering applications, such as those that demand non-invasive control and on-demand functionality.

### XG–PLMA 3D-Printed Hydrogels Combined with PCL MEW Meshes for Producing Hybrid Scaffolds Combining 3D Printing and Melt Electrowriting

For the multiprinting approach combining 3D printing and melt-electrowriting, we first optimized the MEW mesh fabrication by producing 20-, 40-, and 80-layer meshes to assess their stability and reproducibility (Figure 7A). SEM images revealed that increasing the number of layers leads to a greater fiber misalignment (Figure 7A). Scaffolds with 20 and 40 layers demonstrated precise fiber deposition, characterized by well-aligned fibers stacked uniformly. In the 40-layer samples, minor imperfections were observed at the intersection points of the 0°/90° pattern. In contrast, the 80-layer scaffolds displayed significant distortions, disrupting the original grid structure. Thus, as the number of layers increased, the uniformity decreased. Based on these results, 40-layer scaffolds were selected for subsequent experiments, as they provided sufficient stability while maintaining a well-defined reproducible pattern and an average fiber diameter of 38.4 ± 9.9 μm.

**Figure 7:**
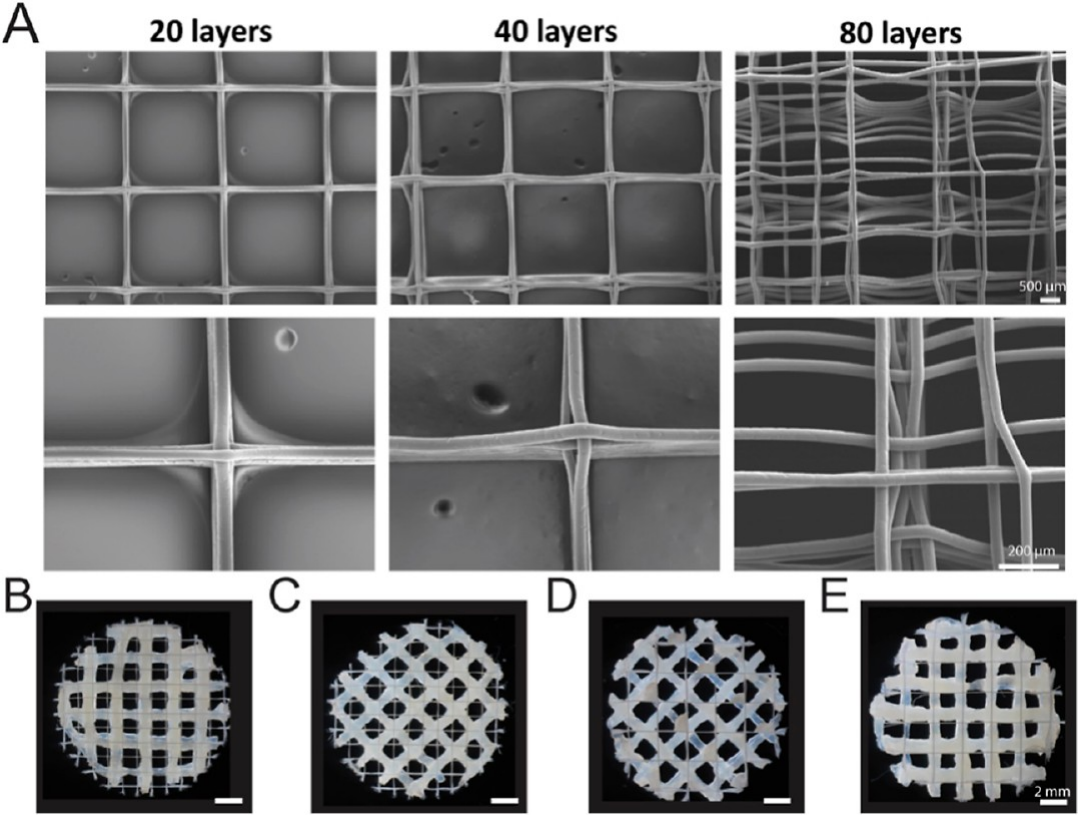
XG–PLMA–PCL hydrogels for a multiprinting approach combining 3D printing and melt-electrowriting. (A) SEM imaging of PCL scaffolds with a 0°/90° 1 mm pitch and 20, 40, or 80 layers. (B–E) Multiprinting scaffolds with different patterns using XG, hPLMA, and PCL (XG5PLMA15 formulation). (B) 0°/90° MEW pattern with 1 mm pitch and 0°/90° PLMA–XG pattern with 2 mm pitch. (C) 0°/90° MEW pattern with 2 mm pitch and 0°/90° PLMA–XG pattern with 2 mm pitch. (D) 0°/90° MEW pattern with 1 mm pitch and 45°/135° PLMA–XG pattern with 2 mm pitch. (E) 0°/90° MEW pattern with 2 mm pitch and 35°/145° PLMA–XG pattern with 2 mm pitch.

To fabricate the hybrid scaffolds combining XG and PCL, we employed a modified 3D Bioplotter (EnvisionTEC) incorporating a high-voltage power supply to fabricate MEW meshes. Thus, our hybrid scaffolds were manufactured in a single piece of equipment in a completely automated manner. First, we identified that manufacturing 40-layer large MEW sheets (70 mm × 60 mm) was more efficient rather than individual single scaffolds due to the relatively slow MEW printing speed (30 mm/s). Automated multiple hydrogel scaffolds were extruded into predefined positions on the MEW sheet. This process showed an improved efficiency and homogeneity in fabricating hybrid scaffolds by combining MEW and 3D printing. Because the hydrogel was 3D printed on the MEW scaffolds, we also explored other combinations among XG–PLMA rather than only XG7PLMA10 to inspect formulations with a higher PLMA ratio (higher bioactivity) and lower viscosities. For that, XG 5% and 7% were blended to PLMA 10% and 15% (XG5PLMA10, XG5PLMA15, XG7PLMA10, and XG7PLMA15). Although these four XG–PLMA variations demonstrated differences in the filament diameter (Figure S17), their flow behavior assessed by viscosity over shear rate and shear strain over shear rate were similar (Figure S18). Hence, a higher ratio of PLMA (from 10% to 15%) and a lower ratio of XG (from 7% to 5%) also held great potential for building hybrid scaffolds, demonstrating the strong XG thickening effect when used both at 5% or 7% (w/v). After MEW and 3D printing processes, the hybrid 3D architectures were punched into circular shapes and double crosslinked, demonstrating stability without layer delamination or collapse. Interestingly, we attempted to 3D print solely hPLMA on the PCL meshes to evaluate the effectiveness of XG in the hybrid scaffold, and no 3D architecture could be obtained (Figure S19), which emphasizes the need for a thickening compound for this material combination.

The alignment and pitch of both MEW and 3D-printed layers were adjusted to optimize the scaffold architecture (Figure 7B–D). One approach involved staggering the MEW mesh position and extruding layers to create interconnected pores (Figure 7B). This effect was further enhanced by combining a 0°/90° MEW pattern with a 35°/145° 3D-printed pattern (Figure 7C,D). Alternatively, matching the angle and pitch of both patterns enabled the direct deposition of the XGPLMA bioink onto MEW fibers (Figure 7E). The final visual appearance and zoomed-in images at the intersection points can be seen in Figure S20. These results demonstrate that the combination of a hydrogel (XG), a source of human bioactive molecules (hPLMA), and a thermoplastic material (PCL) is versatile and can be manufactured in a completely automated manner. These hybrid scaffolds hold great potential to be further explored for complex multimaterial and multibiofabrication approaches that involve several specialized tissues, e.g., in human joints.

## Discussion

Several crosslinking strategies have been developed for XG, but none of them have employed its sole use with cells without prior modification to create biocompatible and stable systems for biomedical engineering applications. Hence, our aim was to develop an ionically crosslinking strategy that would enable the fast and controlled manipulation of the physicochemical and mechanical properties of XG *in situ*, on demand, to be further cultured with cells.

XG hydrogels were ionically crosslinked with several divalent (Ca^2+^, Mg^2+^, and Fe^2+^) and trivalent (Al^3+^ and Fe^3+^) cations through metal–carboxylate ionic interactions and thoroughly investigated *in situ*. We found that XG cannot be ionically crosslinked by divalent cations because it lacks the ability to form the widely accepted “egg-box” junctions between polymer chains and cations.^33^ This result is relevant because some studies have used calcium within XG blends or matrices either for scaffold stability or tablet drug release, respectively.^3,34^ On the other hand, our investigation has shown that XG is prone to form a three-dimensional network in the presence of trivalent cations, via the formation of metal-coordination complexes with high stability. When comparing the stiffness of XG ionically crosslinked with Fe^3+^ and Al^3+^, we identified a 3 times higher stiffness with Fe^3+^ than Al^3+^, indicating that Fe^3+^ cations can form stronger coordination bonds and higher affinity complexes with XG. Fe^3+^ is more electronegative than Al^3+^, thus binding more strongly to XG than Al^3+^. In fact, the same relation among Fe^3+^ and Al^3+^ affinities was also previously reported for crosslinking pectin.^35^

Iron is an essential element in nature, and it is required for numerous cellular processes in the human body.^36^ However, either high or low iron amounts can be detrimental, and several mechanisms exist in our body to balance it within safe limits.^36^ Thus, we herein investigated a large concentration window ranging from 100 μM to 50 mM Fe^3+^ to crosslink XG scaffolds for culturing with hMSCs. Our results have shown the high biocompatibility of hMSCs for 12 days on XG–Fe^3+^ scaffolds, even at the highest concentration (50 mM), and the release of both Fe^3+^ and Fe^2+^ following a smooth and steady trend, without any harmful burst effect. While cell encapsulation is not possible due to the acidic environment required for ionic Fe^3+^ crosslinking, cell infiltration toward the XG scaffolds was noticed 7 days after the cell seeding on XG–Fe^3+^ scaffolds. Moreover, the presence of iron has been previously reported to enhance functionality for tissue engineering applications, e.g., inducing BMP6 (bone morphogenetic protein) expression in hepatic cells^37^ or assisting erythroid differentiation and production of red blood cells via an iron release system based on PEG hydrogels.^38^ Hence, we introduce herein a biocompatible XG–Fe^3+^ system that can be pursued for a wide range of biomedical engineering applications, including tissue engineering, hybrid, and magnetic delivery systems.

The XG–Fe^3+^ hydrogel demonstrated an outstanding printability. Complex printing patterns (Figure 2D), including a challenging Gosper curve (Figure 2E), are often demonstrated in the literature when using thermoplastics,^39^ but rarely with non-modified natural hydrogels. XG–Fe^3+^ also demonstrated high stability for 60 days in culture medium after a single crosslinking step. The currently available non-modified natural hydrogels rarely reach this time point, while synthetic ones are often reported to achieve long stability, e.g., PEG-based hydrogels^40^ and supramolecular hydrogels^41^ for 28 days. In this sense, XG overcomes the limitations of alginate by offering outstanding printability and long-term stability. For the first time, the biocompatibility of the XG–Fe^3+^ hydrogel is demonstrated with human stem cells, with a sustained cytocompatible iron release. Over 12 days in culture, high cell viability and metabolic activity were identified using hMSCs.

XG–Fe^3+^ is an inert hydrogel system that lacks specialized biological receptors to support cell spreading and interactions to enhance cell adhesion, growth, and differentiation in tissue engineering applications. Therefore, to provide bioactive functionality to the XG–Fe^3+^ hydrogel, we introduced hPLMA due to its unique mixture of human proteins and growth factors that can drive tissue regeneration owing to their innate cell-recruiting and pro-regenerative capacities.^24^ For decades, Matrigel has been the gold standard for providing bioactive functionality to inert hydrogels due to its excellent mimicry of the native ECM.^42–44^ However, their tumor origin and complex composition of xenogeneic compounds pose challenges for analysis and clinical applications. hPLMA holds the potential to overcome these limitations as a xeno-free source of growth factors and bioactive molecules, with a very low viscosity that is ideal for blending with viscous hydrogels, such as XG. As expected, cells spread and attached to binding hPLMA adhesive points into the XG–Fe^3+^–PLMA hydrogel, demonstrating enhanced cell adhesion and growth. Yet, hPLMA harmed hydrogel printability, reducing printing fidelity, and increasing the coalescence effect. Hence, XG–Fe^3+^–PLMA gained bioactive functionality but decreased processability compared to the inert XG–Fe^3+^. Finally, the photopolymerizable ability of hPLMA significantly reduced XG swelling, which is often pointed out as a major drawback of XG.^3,5,45^ The ability to control swelling is beneficial for biomedical applications, and herein we introduce two alternatives. When swelling is not intended, e.g., cartilage, joints, and bone tissue engineering, the XG–Fe^3+^–PLMA is the best choice. Alternatively, for biomedical engineering applications where fluids need to be rapidly drained, e.g., post-injury edemas, skin burns, or pulmonary congestion due to excessive fluid accumulation, XG–Fe^3+^ holds greater potential for further exploration.

The ionic crosslinking effect of Fe^3+^ was very pronounced and the mechanical properties of XG–Fe^3+^ were easily tailored to obtain scaffolds ranging from 1.9 to 143 kPa, demonstrating a high versatility for several applications. The highest stiffness value achieved in this work, when using 50 mM Fe^3+^ (143 kPa), is much more superior to other natural hydrogels without prior modification presented in the literature, e.g., decellularized ECMs (generally 25–45 kPa),^46^ gelatin, or hyaluronic acid (generally up to 10 kPa),^46^ or even when using natural blends, e.g., XG and alginate (up to 2.2 kPa)^3^ and alginate and collagen (up to 6 kPa).^47^ However, the Fe^3+^ ionic crosslinking effect also has some limitations regarding pH and solvent employed because an acidic environment is necessary for at least 15 min to obtain the XG–Fe^3+^ crosslinking effect. Also, due to the predominant form of Fe^2+^ in neutral and basic environments, buffer solutions based on divalent ions (e.g., PBS) cannot be used within XG–Fe^3+^ due to the weakening crosslinking effect. Yet, these drawbacks can be overcome by water washes to remove excess iron and by using culture media incubation to neutralize the pH before cell seeding, which maintains the strong and stable XG–Fe^3+^ bond effect for up to two months.

Interestingly, when adding hPLMA into XG–Fe^3+^ and comparing the effect of single ionic crosslinking with a double photoionic crosslinking approach (XG–Fe^3+^–PLMA) using 5 mM, no differences were observed. On the other hand, when comparing 5 to 50 mM Fe^3+^, a great difference was identified. Hence, our results have shown that the Fe^3+^ concentration had a much more pronounced effect on XG mechanical properties than the simultaneous use of two crosslinking strategies. Ionic and photocrosslinking strategies are governed by different chemical interactions. While Fe^3+^ bonds to XG by metal–carboxylate ionic interactions, which is reversible by using divalent ions, methacrylic groups bond to amide groups in hPLMA in a covalent and irreversible (by using common procedures) reaction. Hence, the ability to control the pronounced crosslinking effect (both in XG–Fe^3+^ and XG–Fe^3+^–PLMA), by using a reversible straightforward chemical reaction by reducing Fe^3+^ to Fe^2+^, brings a remarkable versatility to this hydrogel system because the hydrogel can be crosslinked or uncrosslinked to be entirely removed on-demand. Hence, our XG–Fe^3+^ hydrogels demonstrate advantages over others previously reported,^3^ especially for further sensitive assays where the hydrogel must be removed, e.g., for gene and protein expressions.

After demonstrating XG–Fe^3+^ outstanding stability in a culture medium for two months, as well as for carrying cells and human bioactive compounds, we explored its ability to act as a deliverable system that could be guided to the site by external stimuli. When we encapsulated a solution of magnetic nanoparticles (MNPs) into XG, we noticed that the settling velocities of MNPs were reduced due to the network effect.^48^ Due to the gradual stiffness increase of the polymer matrix, particles were trapped in different regions of the XG hydrogel in a homogeneous way. After Fe^3+^ crosslinking, the XG–Fe^3+^–MNP system was able to remotely respond to external magnetic stimulation, performing rotation and translation according to the magnetic operator trajectories. Hence, we have established a remotely responsive scaffold based on the use of a natural polysaccharide and a common element found in the human body, which is utilized both as ions (Fe^3+^) and in magnetic particles (MNPs). The use of remotely stimulated materials is promising for biomedical engineering applications. Some examples of great use of these materials include a blend of starch and polycaprolactone (PCL) for immune modulation,^49^ a blend of PCL microfiber meshes and gelatin-methacryloyl for guiding cell differentiation and tendon regeneration,^50^ a PCL–graphene nanoplatelet composite for guiding the cell organization of skeletal muscles,^51^ and polyisocyanide-based hydrogels to induce changes in cell morphology.^52^ Our XG–Fe^3+^–MNP system differs from the above-mentioned studies in that it uses only natural components to obtain remotely responsive scaffolds. Hence, our system holds the potential for investigation in several applications, especially those requiring noninvasive control and on-demand functionality. Furthermore, magnetic-responsive XG–Fe^3+^–MNP filaments can also be investigated for drug delivery to human tissues that are inaccessible using current medical procedures, such as navigating through long and fragile tubes, e.g., fallopian tubes, or overcoming a ciliated and tiny epithelium (0.5–1 mm), e.g., bronchioles.

Finally, after all XG capabilities were demonstrated as a pure or blended hydrogel, XG–PLMA was employed to create hybrid scaffolds with thermoplastic PCL (XG–PLMA–PCL), offering an alternative for traditional approaches using single materials that have struggled to replicate the complexity of biological tissues. Hybrid multimaterial scaffolds can overcome this limitation by combining materials with complementary properties through the convergence of MEW and 3D printing, leading to improved mechanical performance, durability, and biocompatibility.^53–55^ However, fabricating such scaffolds requires the integration of diverse techniques, each with specific challenges related to material compatibility, resolution, and processing. Synergistically optimizing these fabrication strategies is key to the next generation of engineered scaffolds for tissue repair. Here, two manufacturing techniques were combined in an automated manner in a single additive manufacturing equipment to leverage the advantages of combining thin PCL filaments using MEW and the natural mechanically tunable XG–Fe^3+^–PLMA hydrogel by 3D printing, allowing the manufacturing of hybrid scaffolds.

The resulting XG–Fe^3+^–PLMA–PCL constructs exhibited good layer attachment and stability, ensuring that they can be handled and punched as post-processing steps. Currently, the majority of the strategies for manufacturing multimaterial hybrid scaffolds using combined MEW and 3D printing employ a mesh-filling strategy,^53,56^ where the bioink is confined within the pores of the MEW mesh. While these strategies provide spatial control and enable patterned deposition of different cell types, they restrict free-form printing. Thus, the bioink is often limited to predefined MEW microchambers, resulting in bulky scaffolds that lack microporosity and offer little advantage over conventional MEW and hydrogel casting methods. In contrast, our approach demonstrates accurate free-form printing, allowing for independent design, regardless of the MEW pattern. Using a similar strategy, scaffolds with three levels of porosity (low-micron pores within the hydrogel, micron-scale pores (250–750 μm) in the MEW mesh, and macropores (∼1 mm) in the printed hydrogel were manufactured).^55^ These different pore scales contributed to oxygen and nutrient diffusion, cell elongation and pore bridging, and tissue and vascular ingrowth *in vivo*. Our automated approach, utilizing a single additive manufacturing equipment, ensured high spatial control, with a scaffold resolution relying solely on machine calibration, eliminating manual placement errors. This proof-of-concept on building multimaterial scaffolds leveraging the advantages of natural and synthetic materials and different manufacturing techniques opens new opportunities for developing tailored scaffolds for complex tissue engineering applications, e.g., joints and cardiovascular tissues.

## Conclusion

In summary, we reported that non-modified XG can be effectively crosslinked with Fe^3+^ in an on-demand manner for establishing an XG–Fe^3+^ hydrogel system, with mechanically tunable properties ranging from ∼3 to 203 kPa, remarkable for a natural hydrogel. XG–Fe^3+^ demonstrated an outstanding printability capacity via additive manufacturing techniques and high stability in cell culture environments for up to two months. The XG–Fe^3+^ system is biocompatible with hMSCs culture when seeded on XG–Fe^3+^ scaffolds within Fe^3+^ concentrations ranging from 100 μm to 50 mM. To introduce biological functionality, we blended human methacryloyl platelet lysates (hPLMA) into XG–Fe^3+^ to obtain a bioactive XG–Fe^3+^–PLMA. Under a double crosslinking approach, hMSCs spread and attached to binding hPLMA adhesive points. Hence, XG–Fe^3+^ can be either inert or bioactive (XG–Fe^3+^–PLMA) by carrying cells and bioactive molecules without compromising their mechanical properties. Two proof-of-concept applications were demonstrated. First, we expanded the XG–Fe^3+^ carrier role of cells and bioactive compounds to a delivery role using magnetic nanoparticles (MNPs), and magnetic responsive scaffolds were obtained (XG–Fe^3+^–MNP). Then, XG–Fe^3+^–PLMA was employed for enabling the convergence of 3D printing and MEW, and hybrid scaffolds with PCL (XG–PLMA–PCL) were produced, leveraging the advantages of both natural and synthetic materials in an automated manner. Our results have demonstrated that XG–Fe^3+^–PLMA exhibits remarkable versatility as a natural, mechanically tunable, and bioactive hydrogel, ideal for tissue engineering applications. Moreover, whether used in pure or hybrid scaffolds with PCL, encapsulating magnetic particles or not, we foresee broad applications for biomedical engineering purposes. This combined set of capabilities is rarely reported in other non-modified natural polysaccharides. In conjunction with the current focus on developing tailored scaffolds for complex tissue engineering applications, we foresee broad applications of the XG–Fe^3+^ system, offering a set of raw materials entirely based on natural components.

## Methods

### Preparation of XG Hydrogels

Xanthan gum (XG) from *Xanthomonas campestris* (G1253, lot #SLCC2817, lot #SLCJ4693) was sterilized according to our previously published protocol.^3^ Briefly, the mass of XG in the powder was sterilized by fast autoclaving (pre-vacuum sterilizer, 134 °C) for 4 min. After cooling to 20 °C, the XG powder was mixed with sterile PBS to prepare 2, 5, or 7% (w/v) solutions (XG2, XG5, or XG7, respectively) followed by an intense mixing with a spatula. XG hydrogels were maintained overnight in a roller tube platform to ensure their complete dissolution. For ionic crosslinking of XG, iron chloride hexahydrate (FeCl_3_·6H_2_O), aluminum chloride (AlCl_3_), iron­(II) sulfate heptahydrate (FeSO_4_·7H_2_O), calcium chloride hexahydrate (CaCl_2_·6H_2_O), and magnesium sulfate (MgSO_4_) were used. Before use, crosslinker solutions were prepared by diluting them in Milli-Q water to obtain concentrations ranging from 100 μM to 50 mM, depending on the experiment. All crosslinkers were dissolved in water unless otherwise stated. Scaffolds were ionically crosslinked by soaking in the above-mentioned solutions for 15 min and then washed using Milli-Q water to remove excess unbonded ions.

### Preparation of XG–PLMA Hydrogels

Lyophilized hPLMA was gently dissolved in PBS to final concentrations of 10% or 15% (w/v) solutions (PLMA10 or PLMA15)*.* Next, the ruthenium/SPS photoinitiator system was added following our previously published protocol.^29^ Briefly, SPS and ruthenium were mixed with the previously prepared 10 or 15% hPLMA in a 1:100 v/v ratio to achieve final concentrations of 0.01 m SPS and 0.001 m Ru. These solutions were wrapped in aluminum foil for light protection. Next, the previously sterilized amount of the XG powder to make a 5 or 7% (w/v) solution (XG5 or XG7) was slowly added to the PLMA–Ru–SPS solution while mixing with a spatula and maintained in a roller tube platform overnight to ensure the complete dissolution of XG into PLMA.

### Preparation of XG–MNP Hydrogels

Dried Fe_3_O_4_ nanoparticles (Sigma-Aldrich, 637106) were diluted into PBS to obtain a final 2.5% (w/v) solution. During constant magnetic mixing (500 rpm), the amount of the XG powder to make 2, 5, or 7% (w/v) solutions (XG2, XG5, or XG7) was slowly added to the MNP solution. Next, XG–MNP hydrogels were maintained in a roller tube platform overnight to ensure the complete dissolution of XG into the MNP solution.

### Rheological Characterization

The mechanical properties were analyzed on a Discovery HR-1 rheometer (TA Instruments) using a parallel geometry with a diameter of 20 mm and a gap of 500 μm. The samples were loaded into the rheometer equipped with a cup and equilibrated for 2 min, followed by the addition of 3 mL of salt solutions. Then, a time sweep for 2 min was performed to compare with the mechanical properties before salt addition. The storage modulus *G*′ and loss modulus *G*″ were recorded at a strain amplitude γ = 1% and frequency ω = 1 Hz. Each condition was measured 2 or 3 times to ensure repeatability.

### Temperature Characterization

The effect of temperature variation of XG was assessed using a digital scanning calorimetry (Q2000 V24.11 build 124, TA Instruments, United States) in the range of −90.13 to 397 at 20 °C/min heating.

### Oxygen Measurement in Hydrogels

Oxygen concentrations were assessed using XG–Fe^3+^, alginate–Fe^3+^, and alginate–Ca^2+^ scaffolds. A needle-type fiber optic oxygen microsensor (PSt7, PreSens, Germany) was inserted inside each scaffold and maintained static for over 50 h by coupling it to a micromanipulator (PreSens, Germany).

### Mechanical Testing

XG hydrogels were mechanically characterized by using a TA Electroforce 3200-series multiaxial mechanical tester (TA Instruments). Hydrogel samples for compressive testing were constructed using a custom press mold, allowing for the formation of discs with normalized sample dimensions (D10 mm, H4 mm). This procedure is derived from the compressive testing properties of ISO 604, to optimally standardize the testing procedures. Once formed, the samples were inserted between stainless-steel compressive clamps and subjected to compressive deformation at a strain rate of 1%/s, while the load was recorded using a 1000*g* force (10 N) load cell. Tests were controlled through the Wintest 7 operational software (TA Instruments), where displacement was induced through the vertical displacement of an axial magnet (disp. Δ−6.5/+6.5 mm) and recorded data was plotted in force (Δ*N*) or displacement (Δmm) over time (50 data points/s). Stress/strain curves were calculated by converting force displacement to its respective stress (σ, kPa) or strain (ε, %) over the total area of the sample. The stiffness of the gels was quantified through the compressive Young’s moduli (*E*_c_, kPa), calculated over the linear area of the elastic region.

### Manufacturing of XG, XG–PLMA, and XG–MNP Scaffolds

All hydrogel solutions were loaded into syringe barrels at room temperature, centrifuged at 1000 rcf for 3 min, and coupled to a 3D printer cartridge holder. A tapered tip nozzle with a conical geometry and an internal diameter of 250 μm (25G, 7018391, Nordson) was used in all cases, unless otherwise stated. Prior to use, syringes and nozzles were disinfected by being soaked in ethanol solution (70% v/v) overnight and fully dried. For all hydrogels, printability tests were performed by using a pressure-assisted extrusion technique. For manufacturing XG7 hydrogels, a BioScaffolder 3.1 (Gesim, Germany) was used for manufacturing 10 × 10 mm boxes with a pressure of 0.6 bar and a speed of 50 mm/s using 3 mL syringe barrels. Likewise, a 3D-Bioplotter fourth Generation (EnvisionTEC, Germany) was also employed for manufacturing 30 × 30 mm boxes with a pressure of 0.4 bar and a speed of 20 mm/s using 30 mL syringe barrels. For manufacturing XG–PLMA hydrogels, a 3D-Bioplotter fourth Generation (EnvisionTEC, Germany) was used for manufacturing 20 mm × 20 mm boxes with a pressure of 0.3 bar and a speed of 30 mm/s. For manufacturing XG–MNP hydrogels, a BioX (CellLink, Sweden) was used for manufacturing star-shaped scaffolds with a pressure of 0.4 bar and a speed of 15 mm/s. After printing, all the scaffolds were ionically crosslinked by soaking in FeCl_3_ solution in Milli-Q water for 15 min. For XG and XG–PLMA–PCL scaffolds, a 50 mM FeCl_3_ solution was used, while for XG7–MNP, a 25 mM FeCl_3_ solution was used. For XG–PLMA, a double crosslinking approach was employed by starting with a photocrosslinking step simultaneously with 3D printing (455 nm, 46 mW, 60 s) followed by ionic crosslinking using 5 or 50 mM FeCl_3_ post-printing. For the larger bulk cylinders for mechanical tests, the most similar photocrosslinking parameters were employed using a UVP CL-1000 crosslinker (365 nm, 48 mW, 60 s). After the crosslinking steps, scaffolds were washed twice with water and maintained in water using a constant gentle agitation for 6 h to remove unbound iron in excess.

### Manufacturing of PCL Scaffolds

A modified 3D-Bioplotter (EnvisionTEC) incorporating a high-voltage power supply was used to fabricate MEW meshes. Medical-grade PCL (Corbion, The Netherlands) was loaded into the cartridge and heated at 105 °C. MEW scaffolds (15 × 15 mm) with a 0°/90° pattern, 1 mm pitch, and 20, 40, or 80 layers were fabricated using the following parameters: nozzle size = 25GA; offset = 5 mm; pressure = 0.4 bar; speed = 30 mm; temperature = 105 °C, and voltage = 5.5 kV. The fabricated MEW scaffolds were punched into circular samples (10 mm diameter) using a precision puncher and mounted on aluminum pin stubs with carbon tape. Samples were gold-coated by using a sputter-coater and imaged by scanning electron microscopy (Jeol JSM-IT200 InTouchScope) to assess the morphology. Filament diameters were measured manually by using Fiji.

### Manufacturing XG–PLMA–PCL Hybrid Scaffolds by Combining MEW and 3D Printing

The modified 3D-Bioplotter described above was used for the multiprinting approach, combining MEW and 3D printing. First, 70 × 60 mm MEW scaffolds with 40 layers and 1 or 2 mm pitch were fabricated as described above. Next, 15 × 15 mm XG-PLMA scaffolds with a 0°/90° or 45°/135° pattern and a 2 mm pitch were printed on the MEW scaffold by using the XG5–PLMA15 formulation. The following parameters were applied: nozzle size = 25GA; offset = 1 mm; pressure = 0.2 bar; speed = 30 mm/s; and temperature = 25 °C. The scaffolds were crosslinked for 3 min under 405 nm wavelength at an intensity of 25 mW/cm^2^. Finally, the full constructs were punched into circular samples with a diameter of 10 mm to isolate individual samples from the larger MEW scaffold.

### Cell Seeding and Culture on XG Hydrogels

Human mesenchymal stromal cells (hMSC) at passage 5 (PT-2501, Lonza) were used. HMSCs were expanded by culturing 3000 cells cm^2^ in a T-225 flask until they reached 70% of confluence. A basic medium composed by MEM alpha medium with GlutaMAX (32561-029, Gibco), supplemented with 10% (v/v) FBS (F7524, Sigma-Aldrich), was used. Media refreshments were performed every 48 h. Immediately after washing XG and XG–PLMA scaffolds to remove excess FeCl_3_ during the last manufacturing step, XG and XG–PLMA scaffolds were soaked in the above-mentioned supplemented basic medium overnight. Using a precision punch, circular scaffolds of 5 mm were obtained and placed in 96 nontreated well plates. The cell seeding of hMSC was performed according to a previously published protocol.^57^ Briefly, a cell density of 200,000 cells in 37 μL of the above-mentioned supplemented culture medium, now also containing 10% (w/v) of dextran, was seeded in the middle of each scaffold. The scaffolds were maintained statically in the cell incubator (37 °C, pH 7.2 under a 5% CO_2_ atmosphere) for 4 h with no extra addition of culture medium. After 4 h, they were moved to a nontreated 24-well plate containing 1.5 mL of the above-mentioned supplemented basic medium for each scaffold. Media refreshments were performed every 48 h.

### Cell Viability, Metabolic Activity, Spreading, and Iron Release on XG Hydrogels

To analyze cell viability of the cells on XG scaffolds, live/dead assays were carried out with a staining solution consisting of 2.5 μM ethidium homodimer-1 (EthD-1, E1169, Thermo Fisher) and 1 μM calcein acetoxymethyl (calcein AM, c3099, Thermo Fisher). The samples were analyzed with a fluorescence microscope (Eclipse Ti-ENikon, Japan). To investigate metabolic activity, a Presto Blue cell reagent was used according to our previously published protocol.^29^ To analyze cell spreading on XG hydrogels, histochemical analyses were performed to determine the distribution of cells by staining cell nuclei with DAPI (1:100), and cytoplasm with Alexa Fluor 568-phalloidin (1:100) according to our previously published protocol.^29^ To analyze iron release, the iron assay kit (MAK025, Sigma-Aldrich) was used following the manufacturer’s protocol. Briefly, the media culture supernatant of XG scaffolds cultured with hMSCs were tested to see if they reacted with a chromogen resulting in a colorimetric product (incubated for 30 min, measured the absorbance at 593 nm), which was proportional to total iron (Fe^2+^ and Fe^3+^) present in the sample.

### Scaffold Shape Stability in Culture Medium over 60 Days

XG and XG–PLMA scaffold stability was analyzed for long-period exposure in a culture medium. Four-layered 10 × 10 mm scaffolds were printed and ionically crosslinked by soaking with a FeCl_3_ solution (50 mm, 15 min) and maintained in culture media in the incubator for 60 days. To analyze if the printed shape was maintained and if degradation occurred with time, optical microscopy pictures were taken at days 7, 30, and 60 and compared to those of scaffolds obtained immediately after printing and after crosslinking. Scaffold dimensions were quantified (5 measurements per scaffold, three scaffolds per time point, *N* = 15) using ImageJ to obtain overall area, ratio, and pore size.

### SEM Microscopy

XG hydrogels were freeze-dried and coated with gold in a C150TES sputter coater (Quorum, United Kingdom). Subsequently, images were taken using scanning electron microscopy (SEM, Jeol JSM-IT200 InTouchScopeto, Japan) at an accelerating voltage of 10 kV. Pore dimensions were quantified using ImageJ.

### Statistical Analysis

Statistical analysis was conducted using Prism software (10.2.3 version, GraphPad). Two-way ANOVA was employed with a Tukey’s post hoc comparison to evaluate statistical significance. All assays presented herein were performed in at least three technical replicates. The data are expressed as mean ± standard deviation (SD) values or represented using standard deviation bars in the graphs.

## Supplementary Material





## Data Availability

The data that support the findings of this study are available from the corresponding author upon reasonable request.

## Notes

Magnetically responsive XG-Fe^3+^-MNPs scaffolds. Rotation and translation movements were easily performed with trajectory speed dictated by the magnet orientation (MP4)
Rheological characterization, effect of parameters (pH, temperature, oxygen concentration in XG hydrogels), hydrogel preparation, printability, and cell metabolic activity (PDF)

## References

[1] Varaprasad K., Karthikeyan C., Yallapu M. M., Sadiku R. (2022). The significance of
biomacromolecule alginate for the 3D printing of hydrogels for biomedical
applications. Int. J. Biol. Macromol..

[2] Dávila J. L., D’Ávila M. A. (2019). Rheological
evaluation of Laponite/alginate
inks for 3D extrusion-based printing. Int. J.
Adv. Manuf. Technol..

[3] Decarli M. C., Seijas-Gamardo A., Morgan F. L. C., Wieringa P., Baker M. B., Silva J. V. L., Moraes Â. M., Moroni L., Mota C. (2023). Bioprinting
of Stem Cell Spheroids Followed by Post-Printing Chondrogenic Differentiation
for Cartilage Tissue Engineering. Adv. Healthcare
Mater..

[4] García-Ochoa F., Santos V. E., Casas J. A., Gómez E. (2000). Xanthan gum:
production, recovery, and properties. Biotechnol.
Adv..

[5] Andreopoulos A. G., Tarantili P. A. (2001). Xanthan
gum as a carrier for controlled release of
drugs. J. Biomater. Appl..

[6] Dário A. F., Hortêncio L. M.
A., Sierakowski M. R., Neto J. C. Q., Petri D. F. S. (2011). The effect of calcium salts on the
viscosity and adsorption behavior of xanthan. Carbohydr. Polym..

[7] Kumar A., Rao K. M., Han S. S. (2018). Application
of xanthan gum as polysaccharide
in tissue engineering: A review. Carbohydr.
Polym..

[8] Zhang H. (2017). A comparison of rheokinetics
on static and shear crosslinking processes
of xanthan gum. J. Dispersion Sci. Technol..

[9] Zhang H. (2017). Rheological properties
of water-soluble cross-linked xanthan gum. J.
Dispersion Sci. Technol..

[10] Joglekar M. M. (2020). Crosslinking of gum-based
composite scaffolds for enhanced strength
and stability: A comparative study between sodium trimetaphosphate
and glutaraldehyde. J. Biomed. Mater. Res.,
Part B.

[11] Petitjean M., Aussant F., Vergara A., Isasi J. R. (2020). Solventless crosslinking
of chitosan, xanthan, and locust bean gum networks functionalized
with β-cyclodextrin. Gels.

[12] Zeng C. (2020). Highly biodegradable, thermostable eutectogels prepared by gelation
of natural deep eutectic solvents using xanthan gum: preparation and
characterization. RSC Adv..

[13] Garcia-Cruz M. R., Postma A., Frith J. E., Meagher L. (2021). Printability and bio-functionality
of a shear thinning methacrylated xanthan-gelatin composite bioink. Biofabrication.

[14] Elella M. H. A., Mohamed R. R., ElHafeez E. A., Sabaa M. W. (2017). Synthesis of novel
biodegradable antibacterial grafted xanthan gum. Carbohydr. Polym..

[15] Liu Z., Yao P. (2015). Injectable shear-thinning
xanthan gum hydrogel reinforced by mussel-inspired
secondary crosslinking. RSC Adv..

[16] Le H.-P. (2024). Development of novel iron­(iii) crosslinked bioinks comprising carboxymethyl
cellulose, xanthan gum, and hyaluronic acid for soft tissue engineering
applications. J. Mater. Chem. B.

[17] Kang M. (2019). Characterization of xanthan gum-based hydrogel with Fe3+ ions coordination
and its reversible sol-gel conversion. Carbohydr.
Polym..

[18] Narayanan R. P., Melman G., Letourneau N. J., Mendelson N. L., Melman A. (2012). Photodegradable iron­(III) cross-linked
alginate gels. Biomacromolecules.

[19] Petri D. F. S. (2015). Xanthan
gum: A versatile biopolymer for biomedical and technological applications. J. Appl. Polym. Sci..

[20] Shao H. (2013). Intra-articular injection
of xanthan gum reduces pain and cartilage
damage in a rat osteoarthritis model. Carbohydr.
Polym..

[21] Han G. (2012). Preparation of xanthan
gum injection and its protective effect on
articular cartilage in the development of osteoarthritis. Carbohydr. Polym..

[22] Li J. (2019). Xanthan
gum ameliorates osteoarthritis and mitigates cartilage degradation
via regulation of the Wnt3a/β-catenin signaling pathway. Med. Sci. Monit..

[23] Bernal-Chávez S. A. (2022). Cross-linked polyvinyl alcohol-xanthan gum hydrogel fabricated by
freeze/thaw technique for potential application in soft tissue engineering. RSC Adv..

[24] Santos S. C. N. D. S., Sigurjonsson O. ´. E., Custódio C. D. A., Mano J. F. C. D. L. (2018). Blood plasma
derivatives for tissue
engineering and regenerative medicine therapies. Tissue Eng., Part B.

[25] Santos S. C., Custódio C. A., Mano J. F. (2018). Photopolymerizable Platelet Lysate
Hydrogels for Customizable 3D Cell Culture Platforms. Adv. Healthcare Mater..

[26] Tavares M. T., Santos S., Custódio C., Farinha J., Baleizão C., Mano J. (2021). Platelet lysates-based
hydrogels incorporating bioactive mesoporous
silica nanoparticles for stem cell osteogenic differentiation. Mater. Today Bio.

[27] Monteiro C. F., Custódio C. A., Mano J. F. (2021). Bioengineering a humanized 3D tri-culture
osteosarcoma model to assess tumor invasiveness and therapy response. Acta Biomater..

[28] Sobreiro-Almeida R. (2024). Leveraging Blood Components
for 3D Printing Applications Through
Programmable Ink Engineering Approaches. Advanced
Science.

[29] Caiado
Decarli M., Ferreira H. P., Sobreiro-Almeida R., Teixeira F. C., Correia T. R., Babilotte J., Olijve J., Custódio C. A., Gonçalves I. C., Mota C. (2025). Embedding Bioprinting of Low Viscous, Photopolymerizable
Blood-Based Bioinks in a Crystal Self-Healing Transparent Supporting
Bath. Small Methods.

[30] Chen W., Kumari J., Yuan H., Yang F., Kouwer P. H. J. (2022). Toward
Tissue-Like Material Properties: Inducing In Situ Adaptive Behavior
in Fibrous Hydrogels. Adv. Mater..

[31] Hem, J. D. ; Cropper, W. H. Chemistry of Iron in Natural Water: Survey of Ferrous-Ferric Chemical Equilibria and Redox Potentials; USGS, 1962.

[32] Berstad’ D. A., Knapstad B., Lamvik M., Skjølsvik P., Tørklep K., Øye H. (1988). Accurate measurements
of the viscosity
of water in the temperature range 19.5–25.5 C. Physica A.

[33] Brus J. (2017). Structure and Dynamics
of Alginate Gels Cross-Linked by Polyvalent
Ions Probed via Solid State NMR Spectroscopy. Biomacromolecules.

[34] Groves E., Chaw C. S. (2015). Incorporation of
calcium salts into xanthan gum matrices:
Hydration, erosion and drug release characteristics. Drug Dev. Ind. Pharm..

[35] Günter E. A. (2019). Preparation and properties of the pectic gel microparticles based
on the Zn2+, Fe3+ and Al3+ cross-linking cations. Int. J. Biol. Macromol..

[36] Wallace D. F. (2016). Regulation
of Iron Absorption and Homeostasis. Clin. Biochem.
Rev..

[37] Enns C. A., Ahmed R., Wang J., Ueno A., Worthen C., Tsukamoto H., Zhang A. S. (2013). Increased Iron Loading Induces Bmp6
Expression in the Non-Parenchymal Cells of the Liver Independent of
the BMP-Signaling Pathway. PLoS One.

[38] Brändle K. (2020). Iron Nanoparticle Composite Hydrogels for Studying
Effects of Iron
Ion Release on Red Blood Cell in Vitro Production. ACS Appl. Bio Mater..

[39] van
Kampen K. A., Olaret E., Stancu I. C., Duarte
Campos D. F., Fischer H., Mota C., Moroni L. (2023). Hypotrochoidal
scaffolds for cartilage regeneration. Mater.
Today Bio.

[40] Loessner D. (2010). Bioengineered 3D platform
to explore cell-ECM interactions and drug
resistance of epithelial ovarian cancer cells. Biomaterials.

[41] Hafeez S., Decarli M. C., Aldana A., Ebrahimi M., Ruiter F. A., Duimel H., van Blitterswijk C., Pitet L. M., Moroni L., Baker M. B. (2023). In Situ Covalent
Reinforcement of a Benzene-1,3,5-Tricarboxamide
Supramolecular Polymer Enables Biomimetic, Tough, and Fibrous Hydrogels
and Bioinks. Adv. Mater..

[42] Xu S., Zhao L., Li Y., Gu X., Liu Z., Han X., Li W., Ma W. (2024). Activating the healing process: three-dimensional
culture of stem cells in Matrigel for tissue repair. BMC Biotechnol..

[43] Benton G., Arnaoutova I., George J., Kleinman H. K., Koblinski J. (2014). Matrigel:
From discovery and ECM mimicry to assays and models for cancer research. Adv. Drug Deliv. Rev..

[44] Jang J. M., Tran S., Na S. C., Jeon N. L. (2015). Engineering Controllable
Architecture in Matrigel for 3D Cell Alignment. ACS Appl. Mater. Interfaces.

[45] Nims R. J. (2015). Matrix production in
large engineered cartilage constructs is enhanced
by nutrient channels and excess media supply. Tissue Eng., Part C.

[46] Rastogi P., Kandasubramanian B. (2019). Review of
alginate-based hydrogel bioprinting for application
in tissue engineering. Biofabrication.

[47] Du G., Zhang J., Shuai Q., Li L., Zhang Q., Shi R. (2024). Development of alginate-collagen interpenetrating network for osteoarthritic
cartilage by in situ softening. Int. J. Biol.
Macromol..

[48] Comba S., Sethi R. (2009). Stabilization of highly
concentrated suspensions of iron nanoparticles
using shear-thinning gels of xanthan gum. Water
Res..

[49] Vinhas A., Rodrigues M. T., Gonçalves A. I., Reis R. L., Gomes M. E. (2020). Magnetic
responsive materials modulate the inflammatory profile of IL-1β
conditioned tendon cells. Acta Biomater..

[50] Pardo A., Bakht S. M., Gomez-Florit M., Rial R., Monteiro R. F., Teixeira S. P. B., Taboada P., Reis R. L., Domingues R. M. A., Gomes M. E. (2022). Magnetically-Assisted
3D Bioprinting of Anisotropic
Tissue-Mimetic Constructs. Adv. Funct. Mater..

[51] Cedillo-Servin G. (2024). 3D Printed Magneto-Active Microfiber Scaffolds for Remote Stimulation
and Guided Organization of 3D In Vitro Skeletal Muscle Models. Small.

[52] Chen W., Zhang Y., Kumari J., Engelkamp H., Kouwer P. H. J. (2021). Magnetic Stiffening in 3D Cell Culture Matrices. Nano Lett..

[53] de
Ruijter M., Ribeiro A., Dokter I., Castilho M., Malda J. (2019). Simultaneous Micropatterning of Fibrous Meshes and Bioinks for the
Fabrication of Living Tissue Constructs. Adv.
Healthcare Mater..

[54] Castilho M. (2020). Multitechnology Biofabrication:
A New Approach for the Manufacturing
of Functional Tissue Structures?. Trends Biotechnol..

[55] Ross M. T., Kilian D., Lode A., Ren J., Allenby M. C., Gelinsky M., Woodruff M. A. (2021). Using melt-electrowritten
microfibres
for tailoring scaffold mechanics of 3D bioprinted chondrocyte-laden
constructs. Bioprinting.

[56] Ainsworth M. J., Chirico N., de Ruijter M., Hrynevich A., Dokter I., Sluijter J. P. G., Malda J., van Mil A., Castilho M. (2023). Convergence of melt electrowriting
and extrusion-based
bioprinting for vascular patterning of a myocardial construct. Biofabrication.

[57] Cámara-Torres M. (2022). Effect
of high content nanohydroxyapatite composite scaffolds prepared
via melt extrusion additive manufacturing on the osteogenic differentiation
of human mesenchymal stromal cells. Biomater.
Adv..

