# Supplementary material for: Xanthan Gum–Iron System: Natural, Mechanically Tunable, Bioactive, and Magnetic-Responsive Hydrogels for Biomedical Engineering Applications
Source: ACS Appl Mater Interfaces. 2025 Sep 3;17(37):51588–604. doi: 10.1021/acsami.5c08442 (PMC12447385; doi:10.1021/acsami.5c08442)
Supplement: Supplementary file 2 [file am5c08442_si_002.pdf]

SUPPORTING INFORMATION

Xanthan gum-iron system: natural, mechanically tuneable, bioactive, and magnetic-responsive hydrogels for biomedical engineering applications

Monize C. Decarli[1,2*], Joanna Babilotte[1], Wen Chen[1], Julian Kappesz[1], Tim ten Brink[1], Lisanne Dechant[1], Maria Kalogeropoulou[1], Clarissa Tomasina[1], Catarina A. Custódio[3,4], João F. Mano[3], Lorenzo Moroni[1*]

[1] MERLN Institute for Technology-Inspired Regenerative Medicine, Department of Complex Tissue Regeneration, Maastricht University, Universiteitssingel 40, 6229 ER, Maastricht, The Netherlands.

[2] Department of Biomaterials & Biomedical Technology, University Medical Center Groningen/University of Groningen, A. Deusinglaan 1, AV 9713, Groningen, The Netherlands.

[3] CICECO – Department of Chemistry, Aveiro Institute of Materials, University of Aveiro, Campus Universitário de Santiago, 3810-193, Aveiro, Portugal.

[4] Metatissue, PCI, Creative Science Park Aveiro Region, Via do Conhecimento, Ílhavo, 3830-352, Portugal.

* Corresponding authors: Prof. Dr. Lorenzo Moroni (l.moroni@maastrichtuniversity.nl) and

Dr. Monize C. Decarli (m.caiado.decarli@umcg.nl)

SUPPLEMENTARY FIGURES

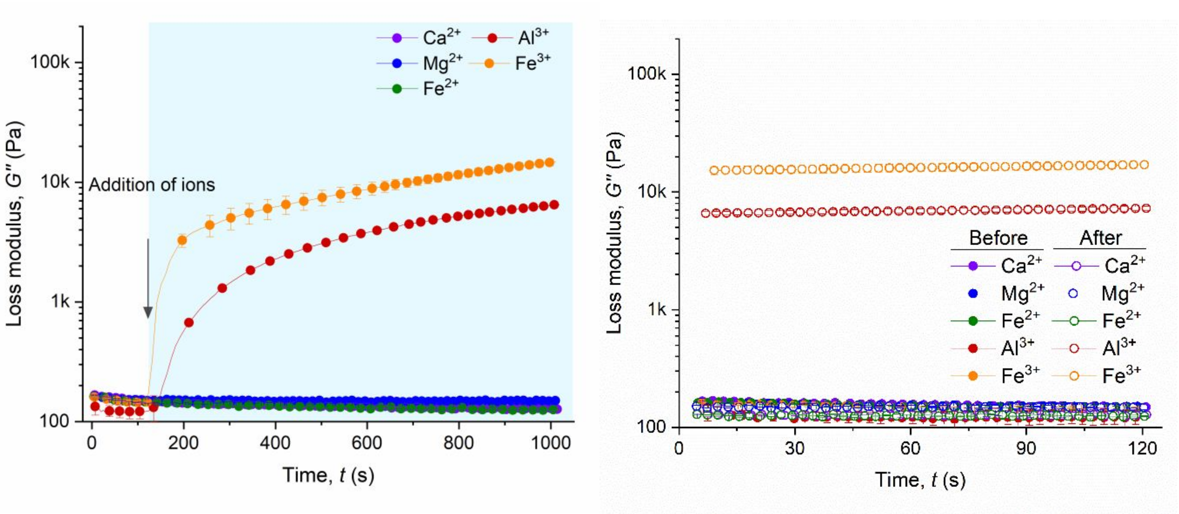

Figure S1. Mechanical properties of Xanthan gum (XG) in the presence of divalent and trivalent cations. *In situ* ionic crosslinking effects of aqueous 50 mM Ca^{2+}, Mg^{2+}, Fe^{2+}, $Al^{3+}$and Fe^{3+} on the loss modulus (G') of 7 wt% XG hydrogel (left) and time sweeps (right) of the loss modulus G' of XG hydrogel before and after aqueous 50 mM Ca^{2+}, Mg^{2+}, Fe^{2+}, $Al^{3+}$and Fe^{3+} addition, using a rheometer equipped with a homemade cup-shaped accessory.

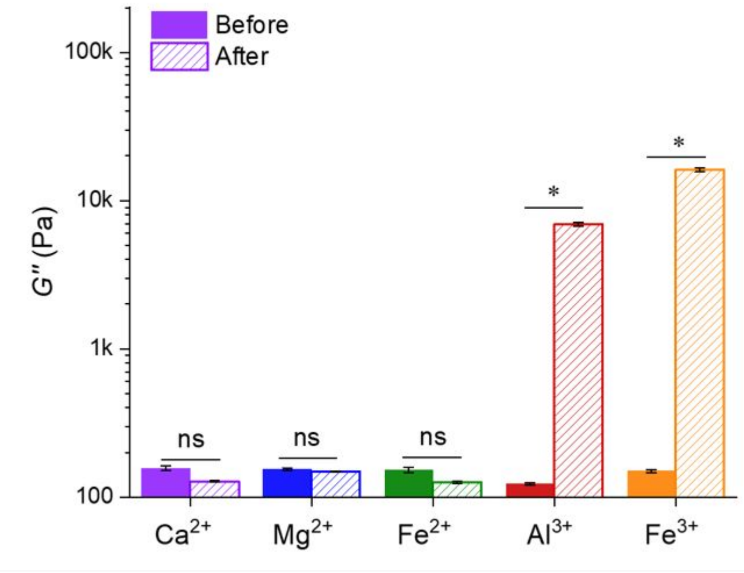

Figure S2. Loss modulus of XG hydrogel before and after aqueous Ca^{2+}, Mg^{2+}, Fe^{2+}, $Al^{3+}$and Fe^{3+} addition, using a rheometer equipped with a 1000 µm geometry gap accessory (significant differences *, $p \leq 0.05$; ns, not significant).

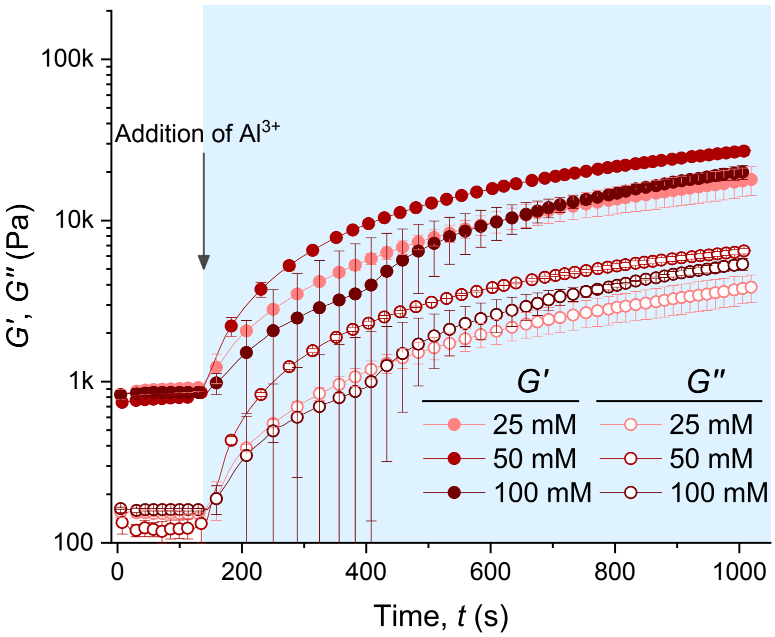

Figure S3. Mechanical properties of 7 wt% XG hydrogels with different concentrations of aluminum chloride dissolved in milliQ water. These measurements were performed using a rheometer with a 1000 µm geometry gap.

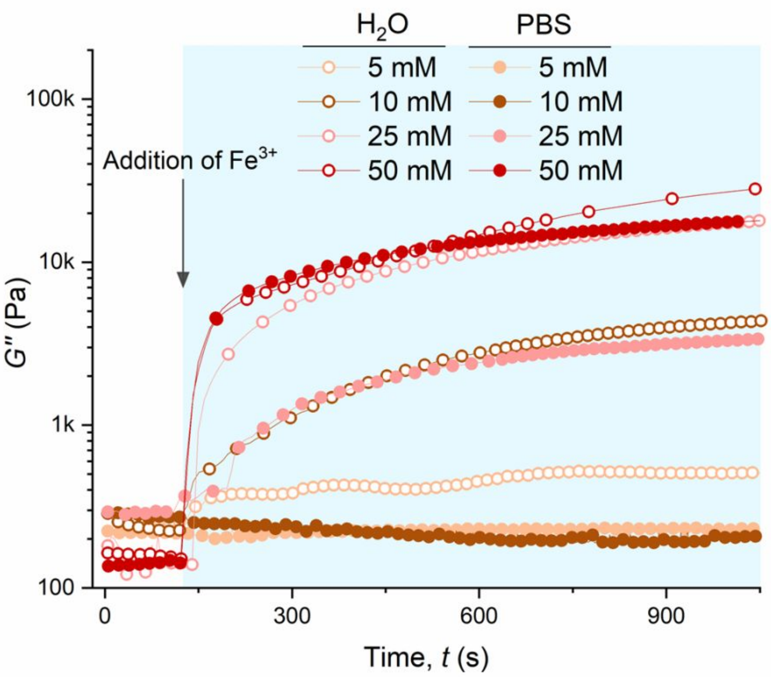

Figure S4. Mechanical properties (loss modulus) of XG ranging from low to high Fe3+ concentrations dissolved in water and in PBS to mimic the conditions when doing cellular culture experiments, using a rheometer was equipped with a 500 µm geometry gap accessory.

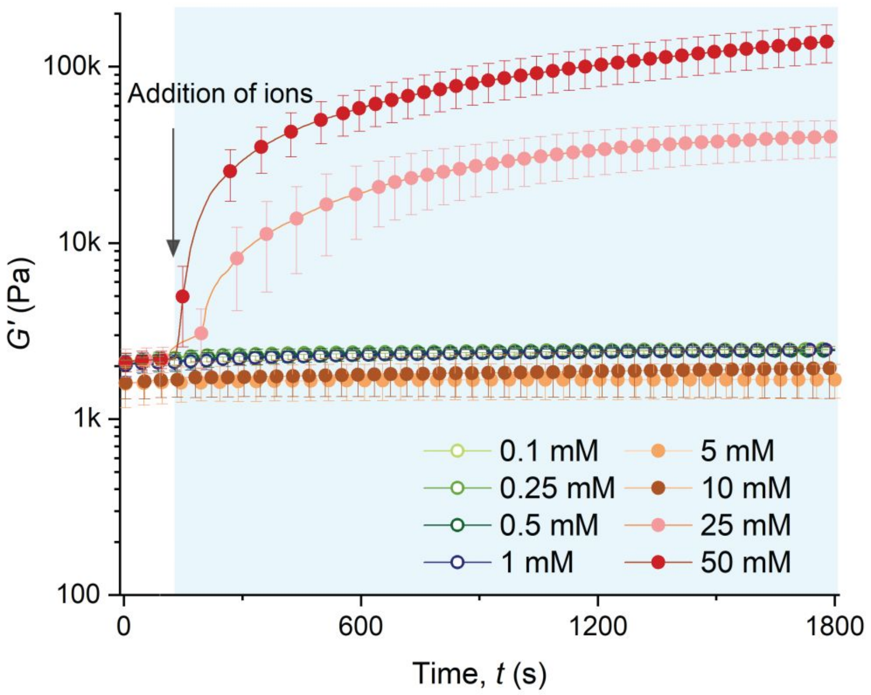

Figure S5: Mechanical properties of XG hydrogels using very low Fe^{3+} concentrations dissolved in water using a rheometer was equipped with a 500 µm geometry gap accessory.

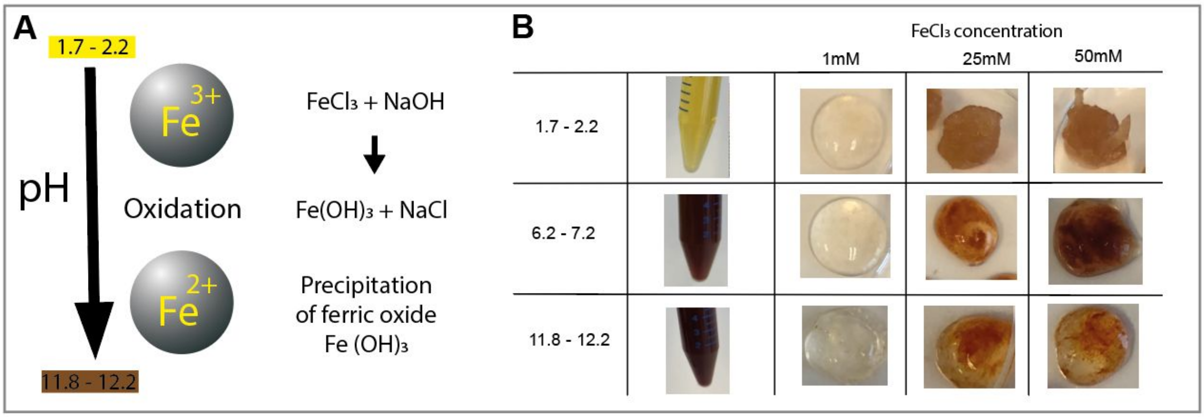

Figure S6. A) An increase in pH by dropping NaOH leads to the precipitation of ferric oxide. **B)** Effect of pH on $FeCl_3$ both in PBS and XG hydrogel solutions. The brown color indicates that ferric iron (Fe^{3+}), initially yellow in acidic pH was reduced to ferrous iron (Fe^{2+}) in neutral or basic pH, thus the crosslinking effect was ruptured.

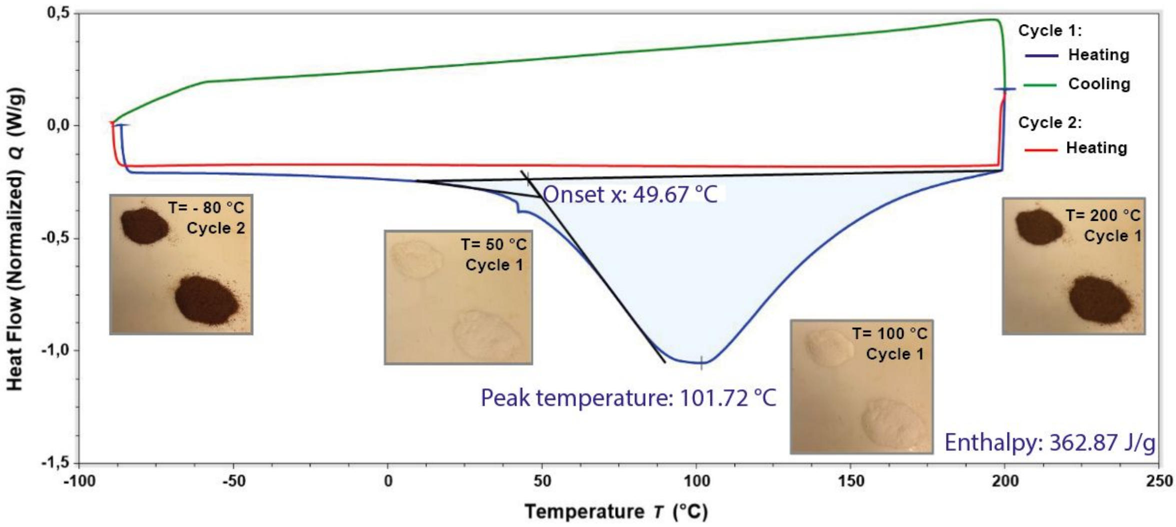

Figure S7. Effect of temperature on xanthan gum, by digital scanning calorimetry. XG is a white opaque powder when stable during heating and it turns brown after thermal degradation.

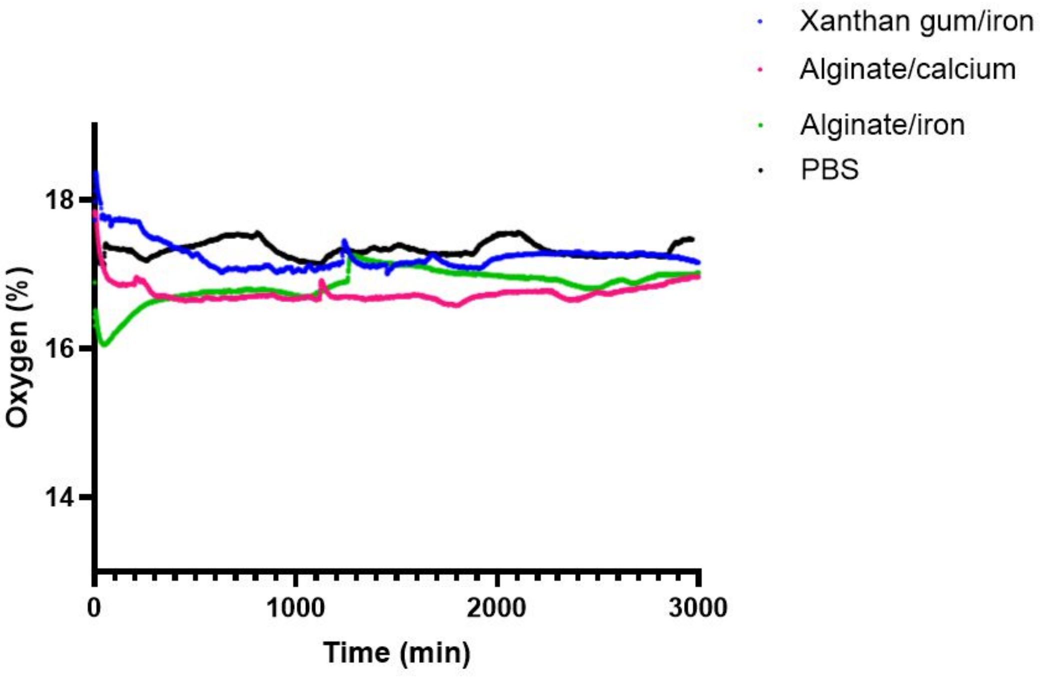

Figure S8. Oxygen concentration assessed in embedded scaffolds (XG-Fe^{3+}, alginate-Ca^{2+}, alginate-Fe^{3+}) and PBS as a control, during 50 hours .

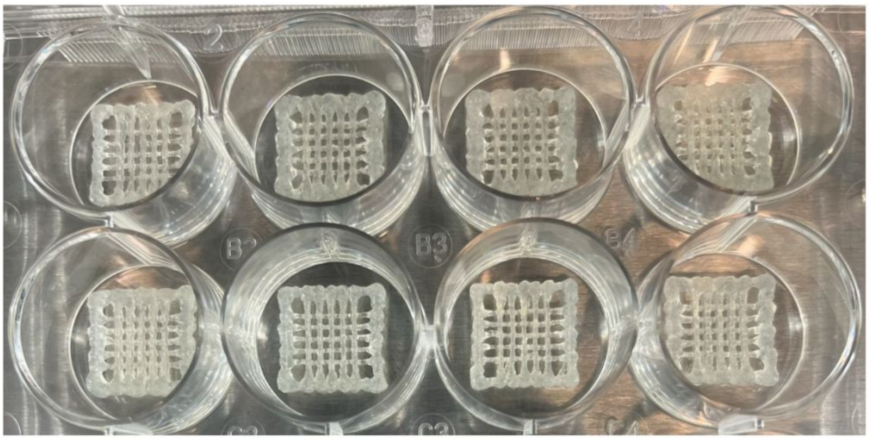

Figure S9. Fast and reproducible scaffolds by a standard extrusion process. Eight scaffolds were produced in 2.5 min (20 s per scaffold).

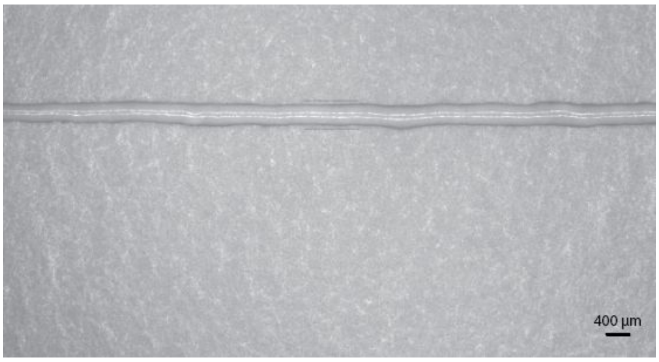

Figure S10. Intact and stable smallest filaments obtained (414 µm), manufactured at a considerably high speed for hydrogels (70 mm/s). Scale bar: 400 µm.

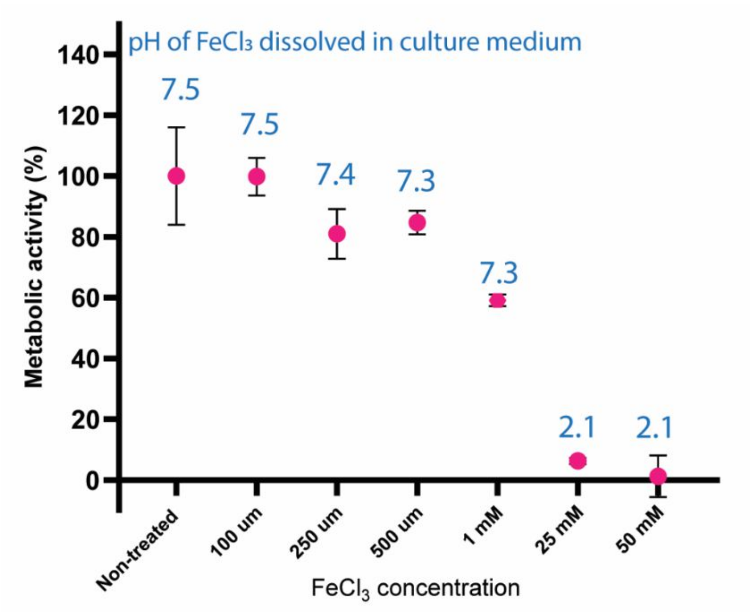

Figure S11. Metabolic activity of hMSCs while increasing concentration of Fe^{3+} due to the acidic environment (pH values of each solution are presented in blue). Non-treated refers to no iron addition (control).

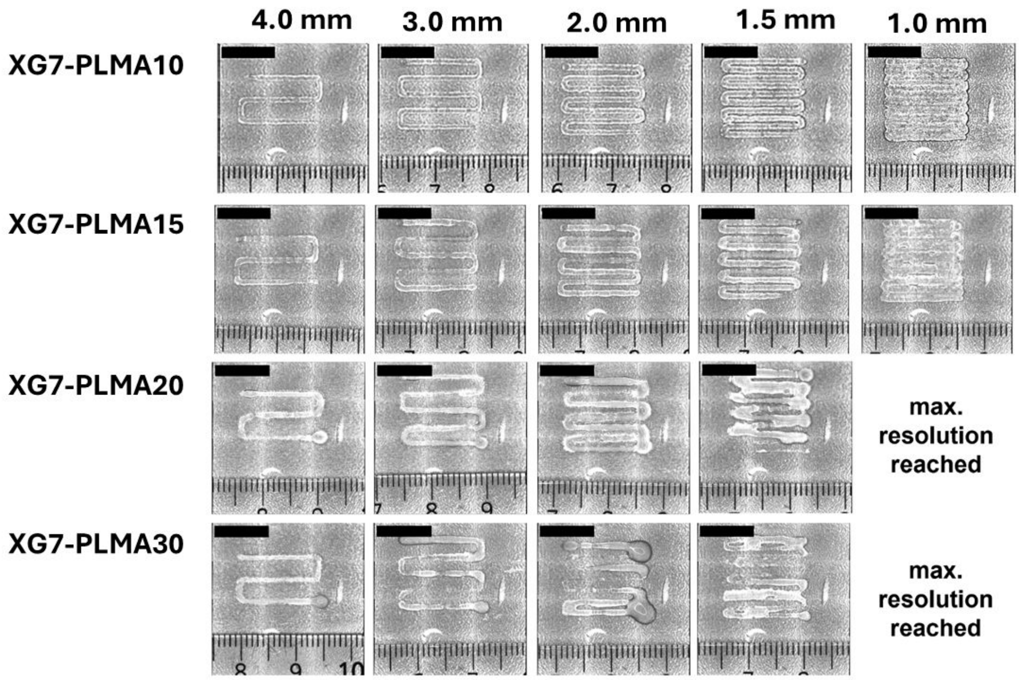

Figure S12: Screening of printable formulations mixing XG 7 % in PLMA solutions made of 10, 15, 20 and 30 % w/v. Printing resolution of the bioinks determined by printing scaffolds with decreasing pitch and visually confirming filament fusion. The pitch in mm is indicated in the top row. Scale bar is 10 mm.

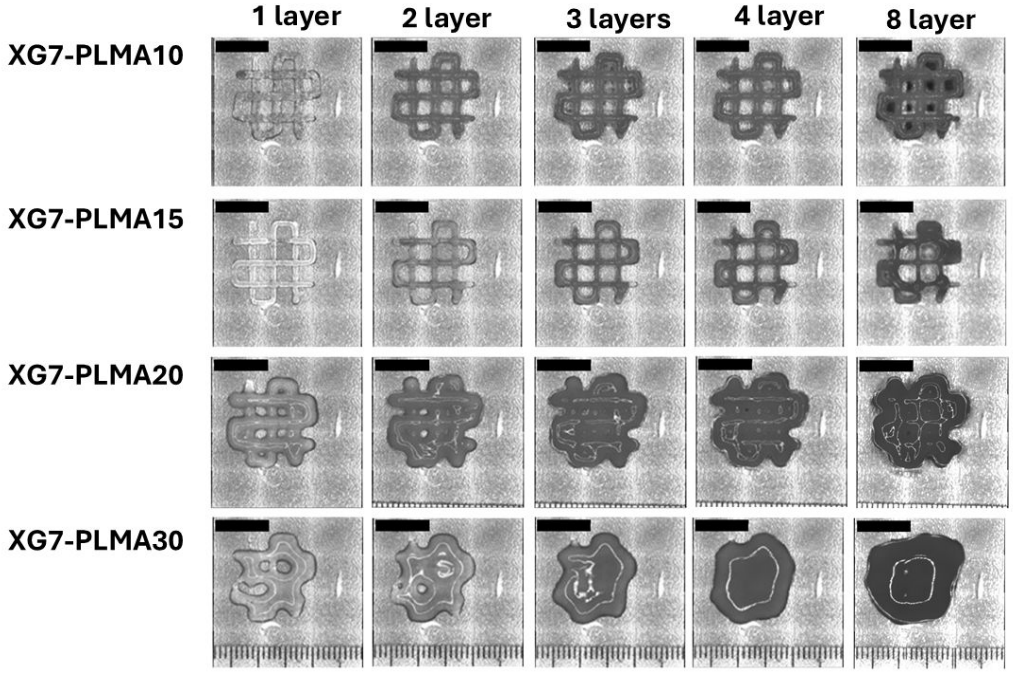

Figure S13: Screening of printable formulations mixing XG 7 % in PLMA solutions made of 10, 15, 20 and 30 % w/v. Printability of multi-layered scaffolds with different bioinks. 20 x 20 mm multi-layer scaffolds with 1, 2, 3, 4, and 8 layers with a 0°/90° pattern and 4 mm pitch directly after printing. Scale bar is 10 mm.

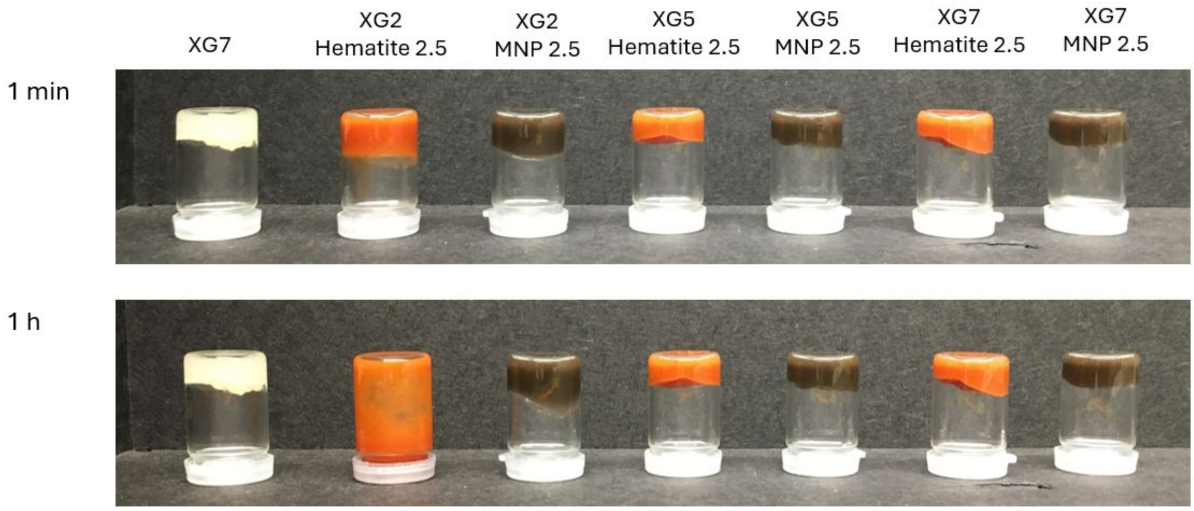

Figure S14. Screening of printable formulations by inversion tube technique after blending XG (2 – 7 % *w/v*) within iron nanoparticles, e.g. hematite and magnetic nanoparticles (MNP).

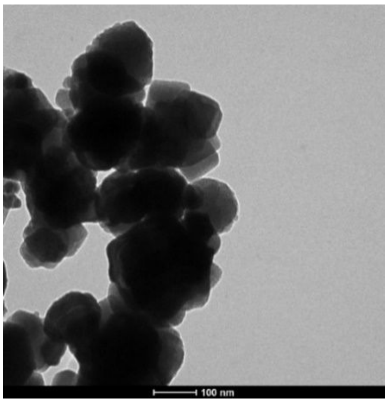

Figure S15. Morphology of magnetic particles (MNPs) encapsulated in XG for soft robotic applications using SEM microscopy.

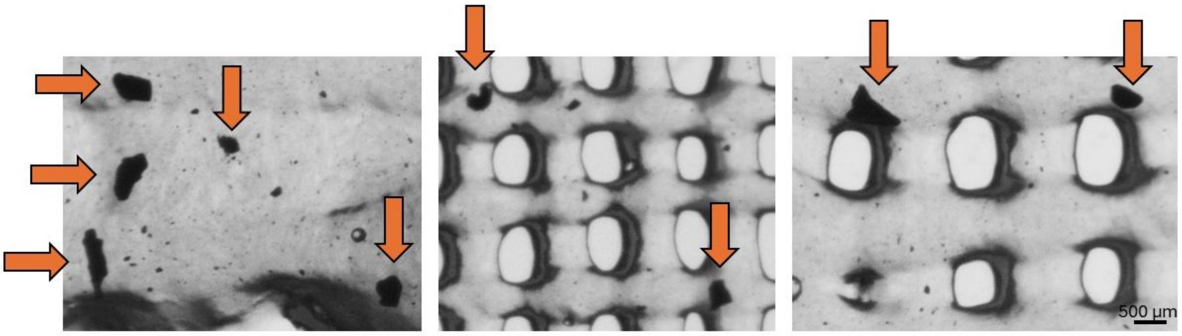

Figure S16. MNPs clustered in some areas of the printed scaffolds to the detriment of others. This happened due to MNP encapsulation into XG hydrogel, instead of prior dissolution in the liquid hydrogel solvent, followed by XG addition.

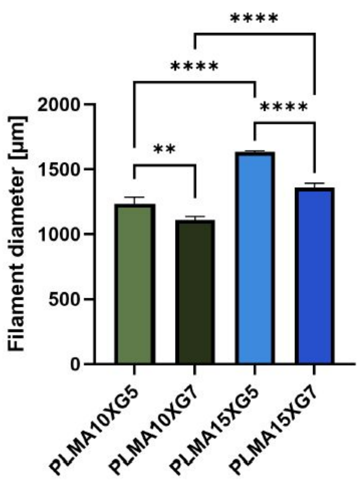

Figure S17. Filament diameter of PLMA10XG5, PLMA10XG7, PLMA15XG5, and PLMA15XG7 formulations.

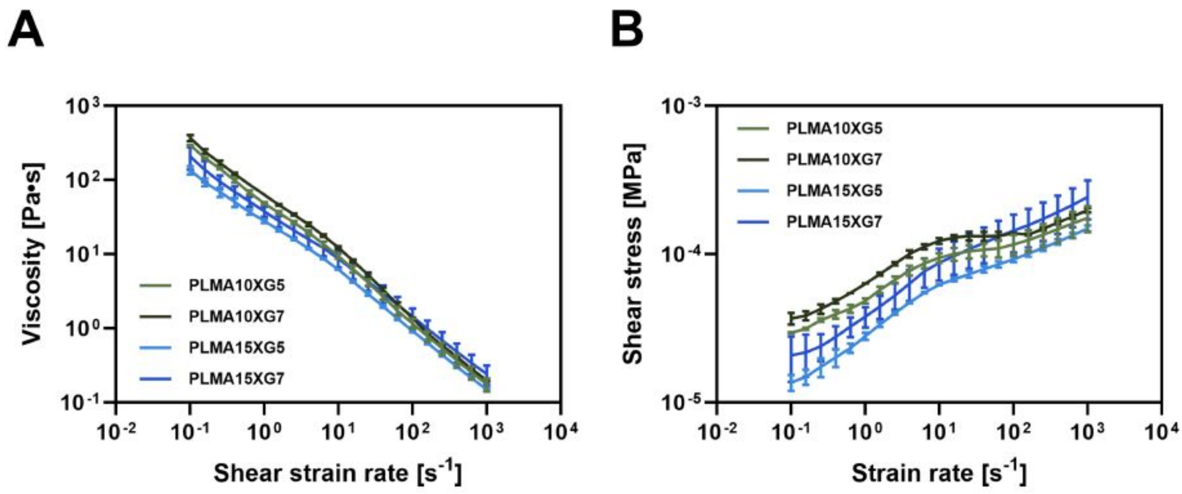

Figure S18: Flow behavior of PLMA-XG formulations (PLMA10XG5, PLMA10XG7, PLMA15XG5, and PLMA15XG7). **A)** Viscosity over shear rate. **B)** Shear strain over shear rate.

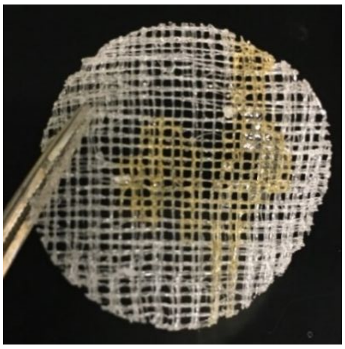

Figure S19: 3D printing of pure hPLMA on PCL meshes to verify the need for a thickener and confirm the effectiveness of XG in the hybrid scaffold. The meltelectrowritten scaffold was punched in a circular shape of 5 mm diameter. The yellow color is characteristic of the Ru/SPS photoinitiator mixed with hPLMA.

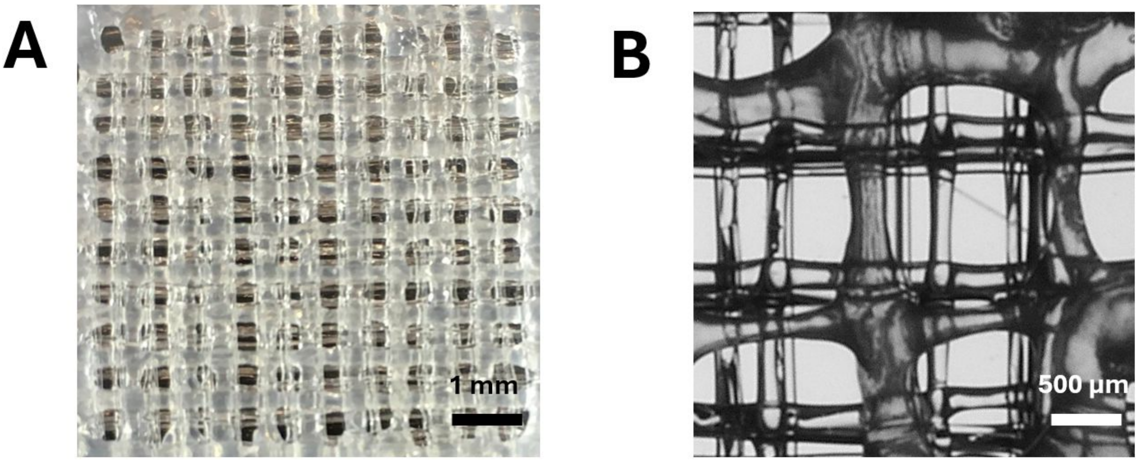

Figure S20: Combination of manufacturing techniques based on 3D Printing the bioactive XG7-PLMA10 on PCL scaffolds produced by melt-electrowriting. **A)** Visualization of the final hybrid scaffolds and **B)** the intersection points of XG7-PLMA10 and PCL in the final hybrid scaffolds.

SUPPLEMENTARY VIDEO

Supplementary Video 1: Magnetically responsive XG-Fe^{3+}-MNPs scaffolds. Rotation and translation movements were easily performed with trajectory speed dictated by the magnet orientation.
